# Legitimacy challenges to the liberal world order: Evidence from United Nations speeches, 1970–2018

**DOI:** 10.1007/s11558-020-09404-y

**Published:** 2020-10-09

**Authors:** Alexander Kentikelenis, Erik Voeten

**Affiliations:** 1 Department of Social and Political Sciences, Bocconi University, Milano, MI Italy; 2 Centre for Business Research, University of Cambridge, Cambridge, CB USA; 3 Edmund E. Walsh School of Foreign Service & Government Department, Georgetown University, Washington, DC USA

**Keywords:** Liberal world order, Global governance, United Nations, Legitimacy, World Bank, IMF, WTO

## Abstract

The liberal international economic order has been facing high-profile legitimacy challenges in recent years. This article puts these challenges in historical context through a systematic analysis of rhetorical challenges towards both the order per se and specific global economic institutions. Drawing on Albert Hirschman’s classic typology of exit, voice and loyalty, we coded leaders’ speeches in the General Debate at the UN General Assembly between 1970 and 2018 as articulating intentions to abandon elements of the order, challenges or calls for reform, unequivocal support, or factual mentions of cooperation. Surprisingly, we find that explicit criticisms towards the liberal order are at an all-time low and that exit threats remain rare. An analysis of the historical evolution of criticisms to global economic institutions reveals a move away from the Cold War insider-outsider conflict towards insider contestation. For example, we find that as countries’ economies become more open, their leaders expressed more support for global economic institutions during the Cold War but less support since. Finally, we demonstrate consistency between the public policy positions leaders announce in UNGA General Debate speeches and their government positions on consequential reform debates on debt relief.

**Electronic supplementary material:**

The online version of this article (10.1007/s11558-020-09404-y) contains supplementary material, which is available to authorized users.

The ongoing deadly pandemic and the looming global economic crisis—both requiring multilateral interventions—arrived at a time when the established liberal international order had already been facing severe strains (Adler-Nissen and Zarakol 2020; Copelovitch et al. 2020; Eilstrup-Sangiovanni and Hofmann 2020; Ikenberry 2018). The global economic order faces challenges from both states that reject its liberal aspirations as well as from liberal democracies in which nationalists and populists have acquired political power. Since 2016, the United States—the most powerful actor in this order—has impeded the functioning of the World Trade Organization’s (WTO) Appellate Body, abandoned the Trans-Pacific Partnership (TPP) trade negotiations, selected a critic of international financial institutions to lead the World Bank, and announced it will cut ties with the World Health Organization (WHO). However, at the same time, countries—including non-liberal regimes and multilateralism-skeptics—are seeking to salvage elements of this order. The EU, China and other countries agreed to a temporary work-around to the impasse at the WTO Appellate Body, the other participants of the TPP negotiations forged ahead without the US, and many countries pledged additional contributions to the World Bank and the WHO. Indeed, no state has recently abandoned global lynchpins like the WTO, World Bank, or International Monetary Fund (IMF).

This is not the first time global economic institutions find themselves in the firing line. To the contrary, challenges to the legitimacy of the liberal order have abounded: calls for a New International Economic Order (NIEO) in the 1970s, major mishandled debt crises in the 1980s and 1990s, the IMF’s disappointing performance in financial crises of the late 1990s and 2000s, the 1999 Seattle protests against the WTO and the aftermath of the global financial crisis that started in 2007 are just some of the most consequential instances. As this list suggests, legitimacy challenges are common, even though the driving forces behind them may change.

This article puts current legitimacy challenges in historical context through a comprehensive analysis of the discourse of governments towards the liberal international economic order in the past half-century. Legitimacy refers to the belief that authority is appropriately exercised within established institutional arrangements (Tallberg and Zürn 2019). Attempts at legitimation or de-legitimation entail justification or contestation of these arrangements (Buchanan and Keohane 2006), which—in turn—shape beliefs. This is a discursive process that takes place in multiple settings and that entails many actors. But perhaps no setting is as symbolically laden as the podium of the United Nations General Assembly (UNGA), where world leaders take the stage every September for the General Debate to deliver their remarks (Binder and Heupel 2015; Boehme 2018; Steffek 2003). We leverage the corpus of speeches for the 1970–2018 period, digitized and made publicly available by Baturo et al. (2017). This setting offers an annual opportunity for leaders to justify or contest the appropriateness of the global economic order, and it also presents an opportunity for social scientists to employ this data to glean patterns and elaborate on a key source of ‘input legitimacy’ to the global institutional order (see Risse 2006; Schmidt 2013).

In examining legitimacy challenges articulated by leaders in the UNGA, our analytical aim is twofold: we study challenges both to the order per se, as well as to its three most prominent organizational underpinnings—the world trading regime (GATT/WTO), the World Bank and the IMF. By liberal international economic order, we understand the post-war system entailing multilateral organizations, open markets, collective responses to policy problems, and hegemonic leadership by the US (Ikenberry 2010, 2011). While the order rests above any single organization, it is upheld by a complex web of such multilateral structures, the most privileged of which being the focal institutions of world trade, development finance, and financial stability (Jupille et al. 2013; Mattli and Woods 2009). In other words, powerful organizations are core elements of the order, while the order is not wholly reducible to them. Indeed, given the global dominance of the order, it affects even countries that are not their member-states or signatories.

To study these issues, we extracted leader statements on the aforementioned topics, and created a coding scheme that draws on Albert Hirschman’s (1970) classic typology of exit, voice and loyalty. We code two types of voice: ‘exit,’ which are clear statements of intent to abandon global economic institutions and ‘challenge/reform,’ which are challenges to and calls for reform of the norms, practices and/or procedures of global economic institutional order. This is a departure from Hirschman’s original argument but justified by the fact that so few actual exits have occurred—instead, our code conceives ‘exit’ as threats or announcements of impending exit, regardless of whether it materializes. We also code two types of loyalty; ‘endorsements,’ which are clear statements of support, and ‘cooperation,’ which are more neutral statements that a country is cooperating with global economic institutions. The resulting data set contains detailed information over a long timeframe on which countries vocalize their criticisms and what reasons they have for challenging the liberal international economic order. In addition, we use automated sentiment analysis to examine the extent to which leader statements use affect to express their positive or negative sentiments.

Empirically, this article offers four core contributions. First, we document that the proportion of speeches that criticize international economic institutions has decreased over time, despite a brief spike around the 2008 financial crisis. Globally, the heyday of critiques of the economic order was the 1975–2000 period for the IMF and the World Bank, and the 1990–2005 period for the world trade system. Respectively, these periods correspond to the rise of demands for a New International Economic Order (mid-1970s until early-1980s) and the ensuing period of the Washington Consensus (after the mid-1980s), and the establishment of the WTO in 1995 and the subsequent Doha Round negotiations starting in 2001. Moreover, we find that both exit threats and explicit endorsements have been scant. Surprisingly, there is only very minor increase in expressions of voice since 2016, and this is outweighed by the increase in statements of loyalty. Indeed, we find that recent years are the only years in which statements of loyalty are more frequent than critical statements, although both are few and far between. More than reflecting notable trends in criticism or loyalty, a defining feature of the past decade is that only less than half the speeches devote any attention to the order: most leaders are simply silent about it.

Second, we present a rich exploratory analysis of the historical evolution of voice against global economic institutions. The textual data allows us to examine changes in the nature of the rhetorical challenges to global economic institutions. Our findings suggest a broader transformation in the nature of contestation away from the Cold War insider-outsider conflict and towards insider contestation. Global economic institutions have long sought to incentivize or compel countries to adopt liberal economic policies, especially with regard to financial and trade openness (Simmons et al. 2008). During the Cold War, many countries openly contested the legitimacy of an international system based on liberal economic principles. Indeed, leaders commonly called for fundamental transformations to the world economic system that would allow their countries more policy space to carve out their preferred developmental trajectories. Since the end of the Cold War, the liberal economic model established near-hegemony and most states are now members of the core global economic institutions. In this context, fewer government leaders contest the ideological foundations of the liberal economic order.

In the 1970s and 1980s, the critiques came primarily from low-income countries that sought to overhaul the entire economic order. In subsequent decades, the overall volume of critical statements declined, and by the 2010s, the proportion of critical speeches by low-income country leaders was approximately the same to those of high-income countries. Moreover, the criticisms were increasingly about particular issues, such as debt or conditionality (i.e., policy reforms attached to World Bank and IMF loans), rather than about the order itself. At the same time, leaders started using their speeches more frequently to signal their cooperation with international economic institutions, even without expressing their support for these institutions. These findings could reflect that lower income countries have started benefiting more from globalization (e.g., Milanović 2016), but it could also be that many governments perceive that the system is immutable and that critique is futile, especially for low-income countries whose voice is likely ignored.

Third, we more systematically examine the implications of this argument. We expect that states that acquired higher levels of trade and financial openness were more supportive of global economic institutions during the Cold War, when contestation was about the desirability of these liberal economic policies. By contrast, in the post-Cold War period, contestation centered more on the impact of these institutions on countries that are moving towards economic openness. Thus, we expect that the more financially open a country becomes the more likely it becomes to critique global economic institutions. We find support for this argument in regression analyses with fixed effects for countries and years. Unlike in the Cold War period, we are now seeing more rhetorical challenges from countries as they open their markets to goods, services and capital, and as they join the core global economic organizations. Thus, while voice has decreased, it is now coming more frequently from countries that have committed to the economic ideology advanced by global economic institutions and that are members of the core organizations.

Fourth, we demonstrate that UNGA General Debate speeches convey meaningful information on underlying state preferences. To do this, we compare leaders’ remarks on debt relief to highly-indebted poor countries—a key issue in global economic governance—with those of two other state officials in more private settings: finance ministers or central bankers attending the IMF and World Bank Annual Meetings (held around the time of the General Debate), and state representatives on the IMF’s board of directors (known as the Executive Board). Most calls for changes to the global debt-management system at the UNGA were repeated or elaborated on by country officials at the World Bank and IMF Annual Meetings. Similarly, country representatives on the IMF’s Board commented in line with their leaders’ stated policy preferences. The main difference was in the use of less affective language in the more private settings of global economic governance, compared to the UNGA.

Beyond these empirical contributions, our findings speak to broader, ongoing theoretical debates within political science. While there is general consensus that the liberal order is facing challenges, there is still much to explain about the actors involved, the arguments they employ, and the venues they use. Indeed, the focus on challenges per se can yield to blind spots about parallel attempts at bolstering and legitimating the order. These processes need not be symmetrical: critiques by some actors in one global forum may elicit defenses by others in a different venue. Globalization and global governance have become a core ideological cleavage in the domestic politics of many countries (Hooghe et al. 2019). Our work points to the need to better understand expressions of both criticism and support, the forms these take, and their ideological underpinnings.

The article proceeds as follows. First, we outline the theoretical foundations of our work, and our data and coding strategy. Subsequently, we analyze broad trends in sentiment towards the liberal economic order. We then examine the characteristics of the countries that engage in criticism and scrutinize its determinants employing regression analysis. The penultimate section draws out the linkages between different global governance arenas to demonstrate that leaders’ UNGA speeches are revealing of state actions in other global fora. We conclude by laying out a research agenda for studying voice against international institutions.

## Discursive legitimation in world politics: The UNGA as a strategic research site

The liberal international order has been remarkably resilient, with relatively limited cases of states exiting organizations or abandoning treaties (Bromley and Meyer 2015; Shanks et al. 1996; von Borzyskowski and Vabulas 2019). This is particularly the case for organizations acting as focal points for global governance (Jupille et al. 2013). Recent evidence on withdrawals from or deaths of international organizations reveal that these events occur primarily for regional, specialized or defunct organizations (Eilstrup-Sangiovanni 2018; von Borzyskowski and Vabulas 2019). For example, exits from the World Tourism Organization, the International Whaling Commission and the UN Industrial Development Organization account for nearly a quarter of all withdrawals from international organizations in the post-war period (von Borzyskowski and Vabulas 2019). In contrast, a closer look at withdrawals from the GATT/WTO, IMF and the World Bank reveals only nine instances, all occurring in the 1950s or 1960s, mostly by socialist countries (von Borzyskowski and Vabulas 2019).

For most states, exit—or deliberate absence—from the liberal international order is not a realistic option, even less so since socialist models disappeared as alternatives (e.g., Gruber 2000; Milanović 2019). The IMF has an effective monopoly as lender of last resort (Lipscy 2016), and alternative arrangements—like the Chiang Mai swap agreement—are small and access usually hinges on a parallel IMF program (Kring and Grimes 2019). Only a few states could realistically use Preferential Trade Agreements to create market access equivalent to that provided by the WTO. There are credible regional alternatives to the World Bank, but even the Asian Infrastructure Investment Bank typically pools resources with the World Bank to fund major projects (Kahler 2017). Consequently, while we can learn much from studying unambiguous and readily-measurable events like withdrawals from international organizations, they only provide part of the story of the legitimacy and staying power—or lack thereof—of international institutions.

Legitimacy is essential to the functioning of most international institutions, and a key building block for its study entails analysis of rhetoric: who says what and why? Discursive challenges to the international order and its constituent elements can undermine legitimacy: the social beliefs that the practices, norms, and procedures of an institution are rightful (Barnett 1997; Bernstein 2011; Best 2007; Hurd 1999; Reus-Smit 2007; Tallberg and Zürn 2019). Consequently, analysis of discourse provides part of the story: it is the ‘missing link’ between ideas about the appropriate course of action and the actions themselves, whether manifested, planned, or contemplated (Schmidt 2008, 2011). Employing such methods has yielded important advances in our understanding of states’ decisions to alter their foreign policies (Schmidt 2017) or our knowledge of how ideas change over time within organizations like the European Union, the World Bank or the IMF (Carta and Morin 2016; Kaya and Reay 2019; Moretti and Pestre 2015). Notwithstanding these contributions, discourse analysis tends to either focus on dyadic issues (e.g., what preferences a country’s leadership expresses vis-à-vis a particular issue) or on the textual output of specific organizations. This is because it is notoriously difficult to study discourse in a systematic cross-national way given its irregular expressions in time, space, form and language.

The UN General Debates offer a unique opportunity to analyze change and continuity in rhetoric by state leaders over a long period in a regularized setting. The UN General Debate Corpus contains the full (officially translated) text of all speeches delivered at the UN’s opening sessions each September between 1970 and 2018 (Baturo et al. 2017). Countries represented by heads of state and governments (45%) get to speak on the first day of the debate, followed by vice-presidents, deputy prime ministers and foreign ministers (45%), and concluding with the heads of delegation to the UN (10%) (Baturo et al. 2017). Each speech is scheduled to last approximately 15 min. The scarcity of time suggests that leaders have to make choices to devote attention to only the most salient issues to them, compared to other, less-pressing topics that they could potentially highlight.

Leaders have long used General Debates to air grievances about the international system (Smith 2003). This can have several uses. First, for many leaders, this is their one opportunity to address a global audience. If a leader uses her limited time to criticize international financial institutions, this may credibly signal dissatisfaction to other governments and institutional leaders. Diplomats of powerful countries reported that their authorities record instances of explicit references to international institutions, like a critique of a multilateral body (authors’ interviews). Officials from international institutions likely also take note when a leader admonishes the institution’s practices, norms, or procedures. In addition, businesses and advocacy groups may pay attention to specific statements, like advertised market-liberalizing reforms to attract foreign investment.

Second, the high-profile nature of General Debate speeches guarantees attention from domestic audiences. Many world leaders are accompanied to New York by media delegations that report on the speech. Consequently, politicians may have political incentives to criticize international institutions if that helps them cater to their supporters at home. For example, political leaders may want to make the IMF a scapegoat for unpopular domestic reforms (Dreher and Gassebner 2012; Vreeland 2003), and the UNGA setting could provide a setting to make such arguments in a global forum.

The diversity of audiences suggests that leaders—and their policy teams—need to craft carefully-worded statements that reflect their country’s priorities and that they take some care on whether or when to adopt strong positions. Clearly, these speeches are not equivalent to global public opinion polls that measure country approval ratings of the liberal international system. Leaders may sometimes use high-profile public speeches to posture for domestic audiences rather than to reveal true policy positions. Further, leaders’ discourse need not translate into more concrete state actions; for instance, a leader might be criticizing international organizations consistently, but not be prepared to exit. But analysis of state actions alone does not illuminate the type and duration of criticisms that states leverage against international institutions.

In sum, General Debate speeches offer important research material for studying the loyalty or challenges to the liberal economic order. After all, the legitimacy of institutional orders is a ‘relational property, determined by the beliefs and perceptions of audiences about the exercise of authority’ (Tallberg and Zürn 2019, p. 586). Knowing who supports or undermines this legitimacy, and how, is important for understanding continuity or change in world politics.

## Legitimacy challenges towards the liberal international order: Context and expectations

The legitimacy challenges faced by the liberal economic order have changed fundamentally over time. The Cold War was characterized by an ideological struggle between capitalist states led by the U.S. and socialist states led by the Soviet Union. Liberal institutions were firmly on one side of that struggle. The core legitimation battle at the global level was between two alternative ideological visions about how the global economic order should be organized and what domestic economic policies should be promoted. Many non-aligned states had reservations about both economic models and about picking sides. This resulted in a North-South cleavage in the United Nations, which cross-cut the East-West Cold War conflict (Kim and Russett 1996; Voeten 2000). Leaders from the global South argued for major distributive structural reforms, whereas leaders from the advanced industrial states advocated for piecemeal reforms through existing multilateral institutions (Doyle 1983). Calls for a New International Economic Order (NIEO) sought to overhaul the global economic system, including a North-South transfer of primary goods, energy, technology and knowledge as well as international taxation, global regulation of multinational corporations, debt forgiveness, and preferential trading arrangements for poorer countries (Bair 2009).

The U.S. and its allies had incentives to placate developing countries whose allegiance in the Cold War conflict was up for grabs. Yet, they also had strong attachments to existing multilateral institutions and the economic order underpinned by these institutions. The U.S. and other advanced industrial states argued that these institutions could benefit developing countries if these implemented market oriented reforms and opened themselves to foreign goods and capital (Doyle 1983). In this Cold War context, we should expect that explicit critiques on the global economic order come from those states that least conform to the liberal economic policies. The exit options to this order entailed either a socialist economic model or the structural changes proposed by the NIEO. Hence, the core legitimation challenge concerned winning the battle of economic ideologies.

The post-Cold War era is different, as the liberal economic model achieved near-hegemony, notwithstanding that countries vary in how they implement capitalism (Hall and Soskice 2001). Even most communist states, with the exceptions of Cuba and North Korea, have switched towards market-based economic management and are well-integrated into the global economic system (Milanović 2019). There are few, if any, plausible exit options based on alternative economic ideologies. For example, the AIIB does not emphasize governance reform to the same extent as the World Bank, yet its economic model is not fundamentally different from the Bank or the two dozen or so other regional development banks (Lipscy 2015b). China’s development model emphasizes a greater role of the state but its growth model is based on an export-oriented market economy, not a fundamentally different economic ideology. Trade agreements also increasingly look alike, even copying much of their text verbatim from other agreements (Allee and Elsig 2019).

Consequently, the legitimation challenge for global economic institutions in the post-Cold War context is less about the fundamental orientation of states towards the economic integration of market-based economies than about whether these institutions manage that integration well. As such, we should expect different kinds of legitimacy challenges that are focused more on specific problems rather than the system as a whole. We may expect challenges from states that are better integrated economically, as these states have more at stake in the functioning of global economic institutions and no longer need to defend these institutions against ideological challengers. That is, we should expect more voice from insiders, countries that have already liberalized their domestic economies, rather than outsiders, countries whose domestic economies are not organized around the principles advanced by the liberal international economic order.

There could be other determinants of variation in how supportive states are of the global economic system. First, there is ample evidence that democracies have been more supportive of especially international trade liberalization, even after accounting for their economic circumstances (Mansfield et al. 2002; Milner and Kubota 2005). States are more likely to join international institutions as they democratize (Mansfield and Pevehouse 2006). Consequently, we expect the attachment of democracies to international institutions to be greater, making them less likely to rhetorically challenge those institutions.

Second, the domestic ideology of governing coalitions matters. Right-wing parties have consistently been more supportive of globalization and trade liberalization than left-wing parties (Milner and Judkins 2004). Therefore, we might expect right-wing governments to be most supportive of global economic institutions. Of course, it is precisely this relationship that appears threatened in the current crisis. Globalization has transformed national political spaces in many countries to make traditional socio-economic divisions less relevant, and has generated new kinds of cleavages (Kriesi et al. 2006; Zürn and de Wilde 2016). The critiques are increasingly coming from ostensibly right-wing governments in democracies. We examine if and how this more recent trend departs from historical practice.

Nevertheless, we expect that during the Cold War, relatively higher levels of economic openness correlate with more supportive statements towards global economic institutions. By contrast, since the end of the Cold War we expect that as countries become more exposed to the international economic system, they become more likely to criticize global economic institutions. The end of the Cold War represents a clear structural break in terms of the average degrees of both financial and trade openness (Chinn and Ito 2008; Gygli et al. 2018). During the Cold War, the very idea of economic openness was contested, and global economic institutions were front and center in that contest. During the post-Cold War period, IFIs have incentivized countries towards more openness than they would domestically prefer, sometimes through harsh reform conditions (Dreher et al. 2015; Kentikelenis et al. 2016). In this context, countries that are moving towards openness and countries that are experiencing financial crises may have more reasons to be critical of global economic institutions.

An auxiliary expectation is that countries experiencing a financial crisis are more likely to critique global economic institutions during the post-Cold War period. The core idea is that critiques in this period are more about the performance of global economic institutions than about economic ideology (e.g., see Stiglitz 2004).

## Exit, voice and loyalty towards the liberal international order: A new data set on discourse in the UNGA general debate, 1970–2018

Albert Hirschman (1970) famously characterized two options for dissatisfied members of organizations: exercise voice in the hope of improving matters or exit as consumers and/or members. Hirschman’s core insight was that opportunities for voice and exit influence each other. When exit is credible, voice may be more effective at influencing reforms. For example, voice has been more effective in reforming the World Bank than the IMF because the exit options for the first are more credible than the latter (Lipscy 2015a). If institutions are responsive to voice, they may prevent exit. Loyalty expressions and silence can reflect true satisfaction with an institution but also a resignation that few credible outside options or effective opportunities for voice exist (Gaventa 1982).

As noted earlier, our analytical objective is two-pronged: examining support for or challenges to the liberal order per se, as well as its institutional embodiments in the form of the world trading regime (GATT/WTO), development finance (the World Bank), and global financial firefighting (the IMF). The order is not solely decomposed into these three institutions, but it permeates their functioning (Ikenberry 2011; Kohli 2020; Ruggie 1982). And solely examining comments about individual institutions can fail to pick up more fundamental sentiment towards the order. After all, absent credible alternatives, it is plausible that countries may simultaneously be members of the WTO or recipients of IMF lending, while also holding deep misgivings about the liberal order at large. Or, conversely, other countries may not be part of the WTO, but nonetheless actively pursue a delegitimation strategy from the outside. In other words, our empirical analysis needs to be able to capture sentiments directed towards both the order and its organizational manifestations.

Employing the corpus of General Debate speeches between 1970 and 2018 (Baturo et al. 2017) and a purpose-built dictionary of terms, we identified all mentions of broad terms associated with the liberal economic order—like ‘liberal order,’ ‘neoliberalism,’ ‘free trade,’ ‘Washington Consensus,’ ‘economic order,’ ‘structural reform,’ or ‘debt management’—and very narrow terms associated with the GATT/WTO, the World Bank and the IMF (variations of their names or references to specific trade rounds). We extracted all text that occurred 50 words before and after mentions.^1^ We developed the codebook and initial sample coding, and then collaborated with graduate students to code the rest of the material—throughout this process we provided support to the coders as necessary (e.g., guidance on ambiguous statements).

Our coding scheme is an adaptation of the Exit, Voice, and Loyalty categorization. Our application of Hirschman’s analytical framework draws on other social-scientific work that has employed it to study diverse issues in international affairs, including discourse on the International Criminal Court (Boehme 2018) or the UN Security Council (Binder and Heupel 2015), US involvement in the League of Nations (Lavelle 2007), and European integration (Slapin 2009; Weiler 1990). To our knowledge, ours is the first analysis to apply this framework to systematically examine state preferences vis-à-vis the liberal international economic order over the past half-century. However, our analytical focus also requires some modifications to Hirschman’s framework that was developed vis-à-vis organizations.

More specifically, we put forward a broad understanding of *Voice*, that includes references to an *intention to exit.* In other words, we code rhetoric about exit rather than actual exits.^2^ In our scheme, this code captures statements that express an explicit intention to abandon an institution, or in other ways suggest that an institution should be abandoned or that countries would be better off not partaking in the institution’s activities. This includes calls for an institution to be replaced by an alternative. Moreover, we include statements by non-member states who explicitly reject joining or call for the abolition or replacement of the core global economic institutions.

We code two additional types of critical voice: *Voice as call for reform* articulates constructive recommendations for changes to the system, like calls for institutions to improve their performance or indications of willingness to engage in discussions about reform. *Voice as challenge* articulates a fundamental criticism or challenge. In our analyses, we combine the two types of *Voice* as reflections of criticism, partially because inter-coder reliability was low in distinguishing between the two.

We also code *Loyalty* in two ways. In its purest sense, loyalty reflects *endorsements* of organizations or the order. This includes efforts to defend it from criticism or calls for countries to better abide by its rules. For example, in 2010 Antigua devoted considerable attention to the favorable ruling by the WTO’s Dispute Settlement Body in its trade dispute with the U.S., and the latter’s unwillingness to comply and offer compensation. The Antiguan leader stated that ‘In good faith, we sought relief within the international system and the democratic principles and rule of law that it is meant to uphold. To be fair, the system delivered justice. But in so doing, its weakness was exposed when justice could not be enforced because the powerful party that was found against would not settle with the small country that was injured.’ Following our coding scheme, this case was coded as loyalty because the underlying negative sentiments were against a violator and not the institution or the order per se—indeed, the country wished the WTO were stronger and does not call for reform nor does it threaten to leave. Further, given our analytical aims, loyalty as endorsement also encompasses cases where a country might not be a member of an organization but may aspire to join and sign up to its core principles. In this context, expressing loyalty is a legitimacy-granting practice, even though that country does not yet have a seat at the table.

In addition, we code the kind of loyalty that does not necessarily reflect affection for an institution, but merely an acknowledgement of *Cooperation and Participation*. These were purely factual statements about collaboration, like implementing a program administered by the IMF. This category is distinct from endorsements, as it is composed of value-neutral comments that merely communicate involvement: for instance, it is possible that a country might silently have misgivings about an organization, while also receiving its financial assistance or seeking posts for its nationals within the organization’s bureaucracy; given ongoing dealings with the organization, the leader might not want to air such grievances at the UNGA. Our coders were reliably able to distinguish factual statements of cooperation from clear endorsements of institutions, so we maintain that separation in our analyses.

Table 1 presents some examples of our coding vis-à-vis the world trading regime, while the Web Appendix contains full coding details. Upon completion of the manual coding, we conducted intercoder reliability checks: inter-coder agreement for all categories (with the merged *Voice as challenge* and *Voice as call for reform* codes, as noted above) exceeded 90%.

**Table 1:** Examples of coding scheme applied to the world trading regime

Voice	as intention to exit	“While we have always supported the principle of free international trade, we expect the new regime to be more realistic and more flexible, to allow ample time for the small and least developed countries to adjust to the new rules. After all, the WTO cannot be considered as a truly global trade regime without the adhesion of all independent countries, including small island States. It is no secret, as the Vanuatu Prime Minister clearly stated at the recent G-77 Summit in Havana, that the conditions being imposed on us for joining the WTO are simply beyond our capacity to consider in the short to medium term. Unless the powerful countries review their positions and conditions with regard to our application, then the Republic of Vanuatu will have no other choice but to reconsider its original application to join the WTO.” – Vanuatu, 2000
	as challenge	“… the protectionist measures adopted by the developed countries and the disregard of the fundamental interests of the developing countries in multilateral trade negotiations have led to a situation in which the results have recently been even more unsatisfactory than before. This is in flagrant contradiction with the commitments and decisions contained in the Tokyo Declaration and the repeated declarations of the developed countries regarding the need to have an open trade system favourable in particular to the developing countries. We are particularly concerned at the tendency to apply the rules of GATT, to which third-world countries must adjust.” – Guinea-Bissau, 1979
	as call for reform	“The new international trade regime has put small States at a disadvantage with the expectation that they liberalize their markets and open their borders in conformity with the obligations and commitments of the World Trade Organization. The playing field must be levelled so that due regard can be given to the fragile domestic economies of these States and their macroeconomic position. If the developed countries are demanding protection for their sensitive domestic industries, then similar demands by developing countries with respect to their domestic industry should not be viewed as a request that is unreasonable or inconsistent with current international trends.” – Bahamas, 1997
Loyalty	as cooperation and participation	“Every sixth Member of the United Nations is a landlocked developing country whose remoteness from world markets and high transport costs are a major impediment to its development. Along with other members of this group, Mongolia is endeavouring to advance our common interests at the United Nations and in the World Trade Organization.” – Mongolia, 2011
	as endorsement	“The year 2013 will mark a turning point for European economies. The Czech economy is highly dependent on international trade, and I strongly believe that free and fair trade is one of the best tools for improving the world economic situation. The multilateral approach to trade issues should be revitalized and cooperation within the World Trade Organization (WTO) reinforced. I sincerely wish much success to the new WTO leadership.” – Czech Republic, 2013

The Appendix is available on the Review of International Organizations' webpage

In addition to this hand-coding, we use an automated sentiment analysis (Rinker 2020). The approach we use, implemented in the R package Rsentiment, improves on naïve dictionary-based bag of words approaches by taking into account ‘valence shifters,’ such as negators (e.g. ‘*not* approve’), amplifiers (e.g. ‘*strongly* approve’), de-amplifiers (e.g. ‘*hardly* approve’) and adversative conjunctions (e.g. ‘approve *but*’). Sentiments are estimated at the sentence level and then aggregated to the statements we extracted from the speeches.

This sentiment analysis serves two purposes. First, the sentiment analyses show that the broad patterns we uncover in the data are robust to automated coding. A plausible future extension of this project is to use machine learning methods to code statements critical of international institutions. Second, we examine the extent to which there is temporal and cross-institutional variation in the amount of affect used in statements. Sentiment analysis can enrich assessments of whether a statement is critical of an institution by measuring the extent to which statements use words that express strongly negative or positive emotions. Scholars have suggested that affective language may play an important role in legitimizing or delegitimizing international institutions (Busch and Pelc 2019).

**Fig. 1:**
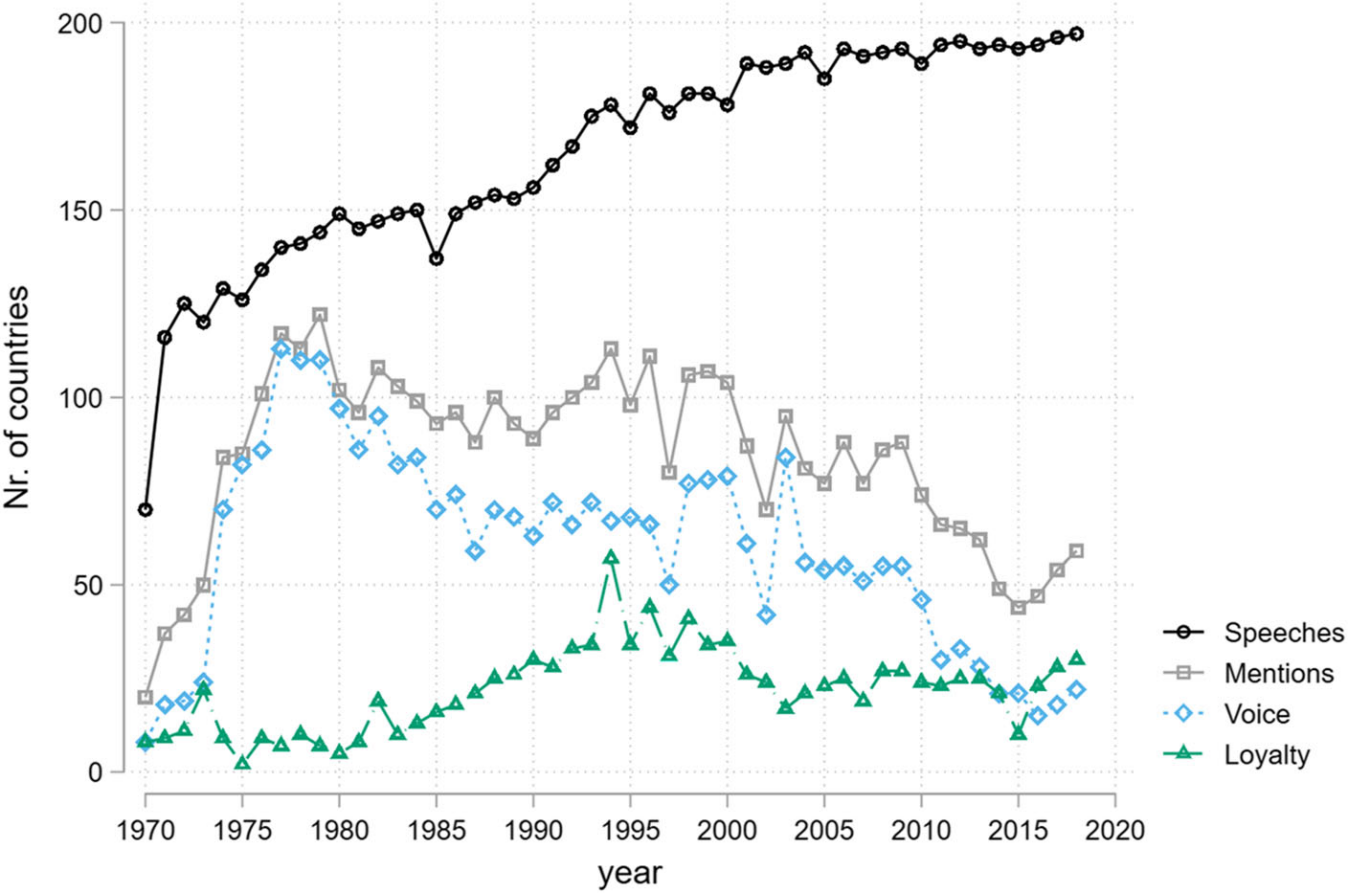
Number of speeches in UN General Debates that express voice and/or loyalty towards the liberal economic order and its institutions

Overall, we examined a corpus of 8093 speeches, 51% (4126) of which discussed at least one of the economic institutions in our dictionary. Half of these speeches mentioned global economic institutions multiple times. Not all of these mentions expressed a clear sentiment that fit our coding scheme. For example, some mentions of ‘tariffs’ were not targeted against the GATT/WTO. In total, 2923 speeches contained some form of criticism towards economic institutions, whereas 1067 speeches contained more cooperative mentions of economic institutions. Some of these (323) contained both critical and more cooperative statements. Figure 1 shows that there is considerable temporal variation in the number of states holding speeches as well as in the number of speeches that mention and that express voice (including exit threats) or loyalty towards global economic institutions. Even as the number of speeches in the General Debate has increased with the number of states in the international system, the number of speeches that mention or express sentiment towards global economic institutions has decreased since 2000. This is almost entirely due to a decline in voice. Indeed, the years since 2016 are the only years when expressions of loyalty outnumber rhetorical challenges to the global economic institutional system.

Before discussing these patterns in more detail, we note the evidence of increasing silence towards the liberal world order: until the 1990s the majority of speeches mentioned the order or its institutions at least once, while the 2000s witnessed a sharp decline in the proportion of speeches making such references. However, after 2015 we observe a slight uptick in such mentions. Interpreting silence poses important analytical difficulties, and we reflect on these issues—and their implications for the legitimacy of the order—in the concluding section.

## Rare threats of exit, declining voice, low-level loyalty

**Fig. 2:**
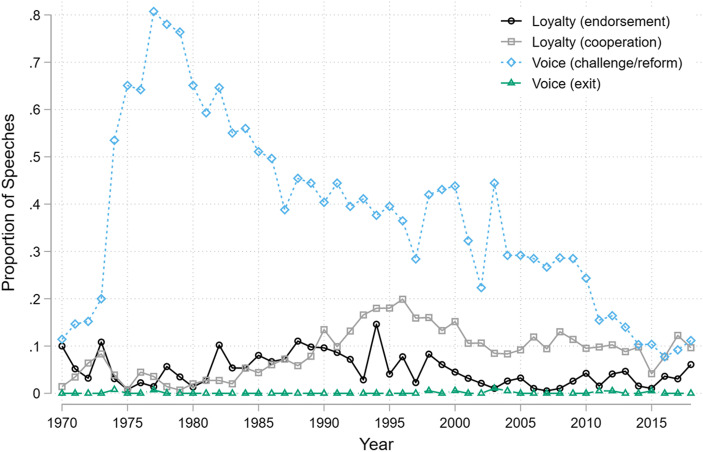
Loyalty and Voice towards the liberal economic order and its institutions in the UNGA General Debate. **Note:** The figure combines Voice as challenge and Voice as call for reform into a single category because of low inter-coder reliability in distinguishing between the two.

Figure 2 summarizes the valence of comments at the General Debate since 1970. First, very few leaders use their 15 min to announce their intention to abandon established forums or call for their wholesale replacement. Of course, there are exceptions: in particular, leftist Latin American leaders with strong ideological objections to the liberal international order are strongly overrepresented among heads of state using the UNGA platform to call for exit, including appeals by Venezuela’s Hugo Chavez, Nicaragua’s Daniel Ortega, Cuba’s Fidel Castro, and Bolivia’s Evo Morales. For example, following a trenchant critique of IFIs and the Washington Consensus, Chavez called for mass resistance to established global economic arrangements:

‘In my country, by the late eighties, a set of structural adjustments, developed under the influence of the main centres of neo-liberal capitalism, was met with a popular uprising that paralysed the country, leaving an indelible mark on our people’s minds. … [T]hat event provoked the necessary consciousness, igniting a political awakening among the people that allowed them to unite their voices in the fight against neo-liberalism. Over the next decade, we witnessed protests against the World Trade Organization and against neo-liberalism in Chiapas, Davos, Seattle, Prague, Quebec City and Genoa. Wherever the architects of neo-liberalism gathered, they were met with massive protests in the streets. … What we need to decide now is whether we will march in the streets alongside our people or hide ourselves away in an ivory tower. My fellow leaders, have we no eyes and ears? Can we not see the suffering? Can we not hear the cries of the poor, the disenfranchised, the disappeared and the desolate?’

Second, leaders also do not often express unambiguous support (endorsements) for economic institutions. The examples mostly come from expected corners. For instance, in 2017, Singapore’s foreign minister emphasized that the WTO was ‘indispensable,’ and Norway’s UN ambassador explained that ‘it is vital that we demonstrate our shared commitment to a rules-based, multilateral trading system, with the World Trade Organization at its core.’

Third, statements of cooperation and participation have increased over time. In the past decade, approximately 10% of speeches refer to collaboration with economic institutions. For example, in 2017 Georgian prime-minister Giorgi Kvirikashvili announced all the reforms Georgia was undertaking in the hope of EU membership, including having ‘agreed to a multi-year programme with the International Monetary Fund and […] secured financing for projects worth several billion dollars from international financial organizations.’ These factual statements do not express specific sentiments towards the institutions in question, but reflect leaders’ attempts to showcase their countries as active and cooperative members of the world polity that seek and achieve participation in high-profile international bodies or are collaborating with them. Leaders might emphasize collaboration with the IMF or the World Bank as a mode of virtue signaling to an international audience. Indeed, our data suggests that virtue signaling towards economic institutions primarily takes the form of communicating cooperation rather than unambiguous support: there are 193 speeches that announce a country’s cooperation with the IMF and 77 expressions of loyalty. In particular, since 1990 it has become more common for countries under an IMF program to express that they are cooperating with the IMF than to voice criticism of the institution.^3^ By contrast, prior to 1990 countries with an IMF program were more likely to criticize the institution.

Finally, a high proportion of leaders voiced criticisms and challenges towards international institutions. Importantly, critiques rapidly escalated in the early-1970s, in the aftermath of the collapse of the Bretton Woods system of fixed exchange rates and the 1973 oil crisis—combined, these events created important balance-of-payments problems for many developing countries (Fioretos and Heldt 2019; Helleiner 2019). In May 1974 the UNGA adopted a resolution proclaiming the establishment of a New International Economic Order, and—a few months later—we observe a large increase in the proportion of General Debate speeches critical towards the global economic order: an increase from 20% in 1973 to over 50% in 1974. NIEO proponents advocated for a North-South transfer of primary goods, energy, technology and knowledge as well as debt forgiveness, and preferential trading arrangements for poorer countries (Bair 2009).^4^ Such critiques peaked during the end of the 1970s, and deescalated over the 1980s as developing countries became embroiled in deep crises that—in marked contrast to NIEO ambitions—made them more dependent on Northern countries and liberal multilateral institutions. After the end of the Cold War, criticisms towards the international economic order further declined, reaching historically low levels in the 2010s.

**Fig. 3:**
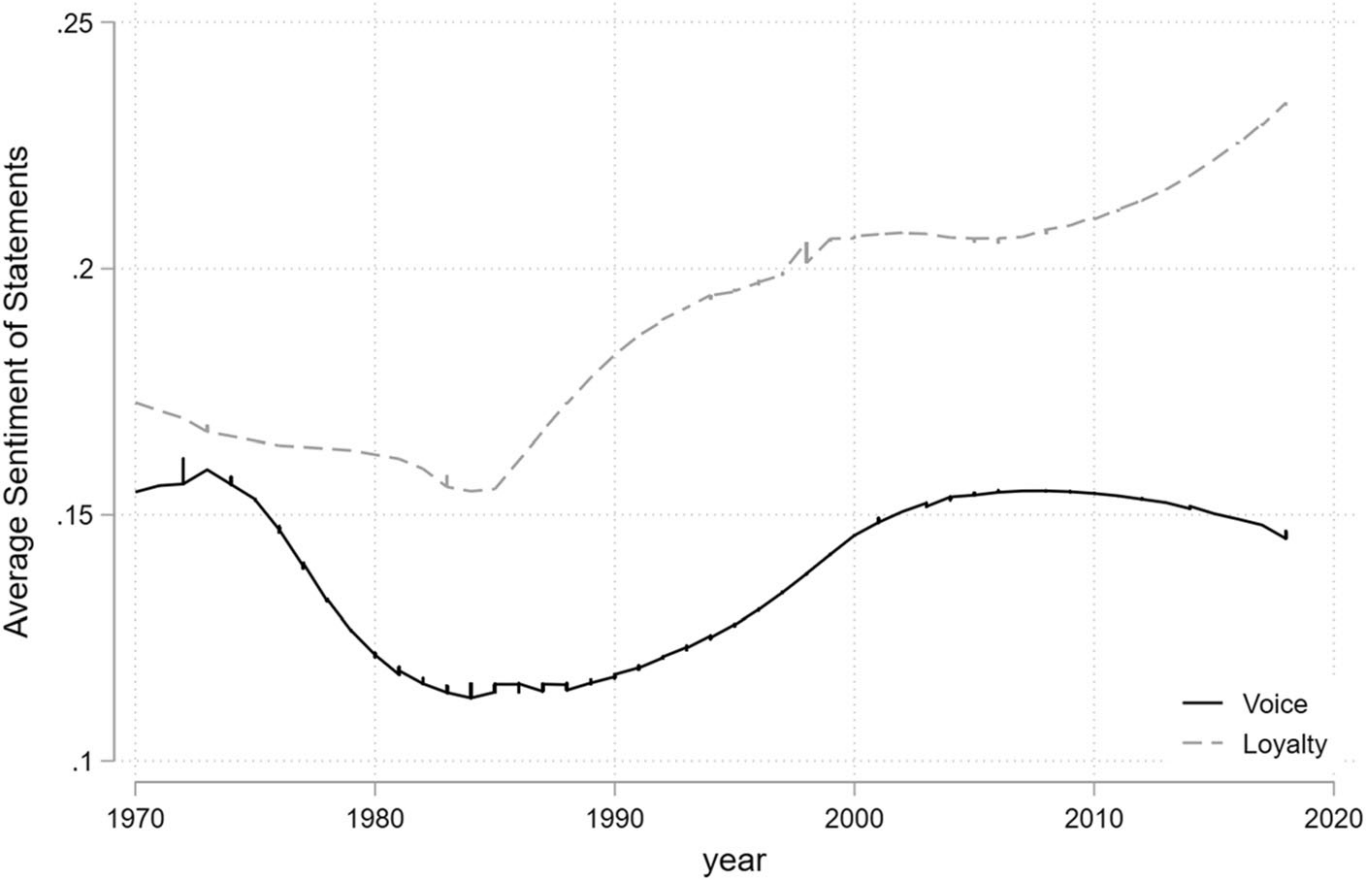
Lowess curves of sentiments expressed in UNGA statements towards the liberal economic order and its institutions

Figure 3 plots a lowess trend in the average sentiment scores for loyalty (endorsement and cooperation) and critical (voice as intention to exit, as challenge or as call for reform) statements towards global economic institutions.^5^ As described earlier, the sentiment scores were computed from the coded excerpts using the R package sentiment. Lower scores indicate more negative sentiment, with negative scores indicating more negative than positive sentiment words. The statements have an average sentiment score of .16 and a standard deviation of .14 (minimum score is −.42, maximum .81). On average, critical statements are about half a standard deviation more negative than cooperative statements. Importantly, the most negative sentiment was expressed during the 1980s. Since the end of the Cold War, even critical statements express less negative emotion, as captured by our sentiment analysis. This supports our findings from Figs. 1 and 2 that the volume of critical statements has been sharply declining.

## Voice about what?

**Fig. 4:**
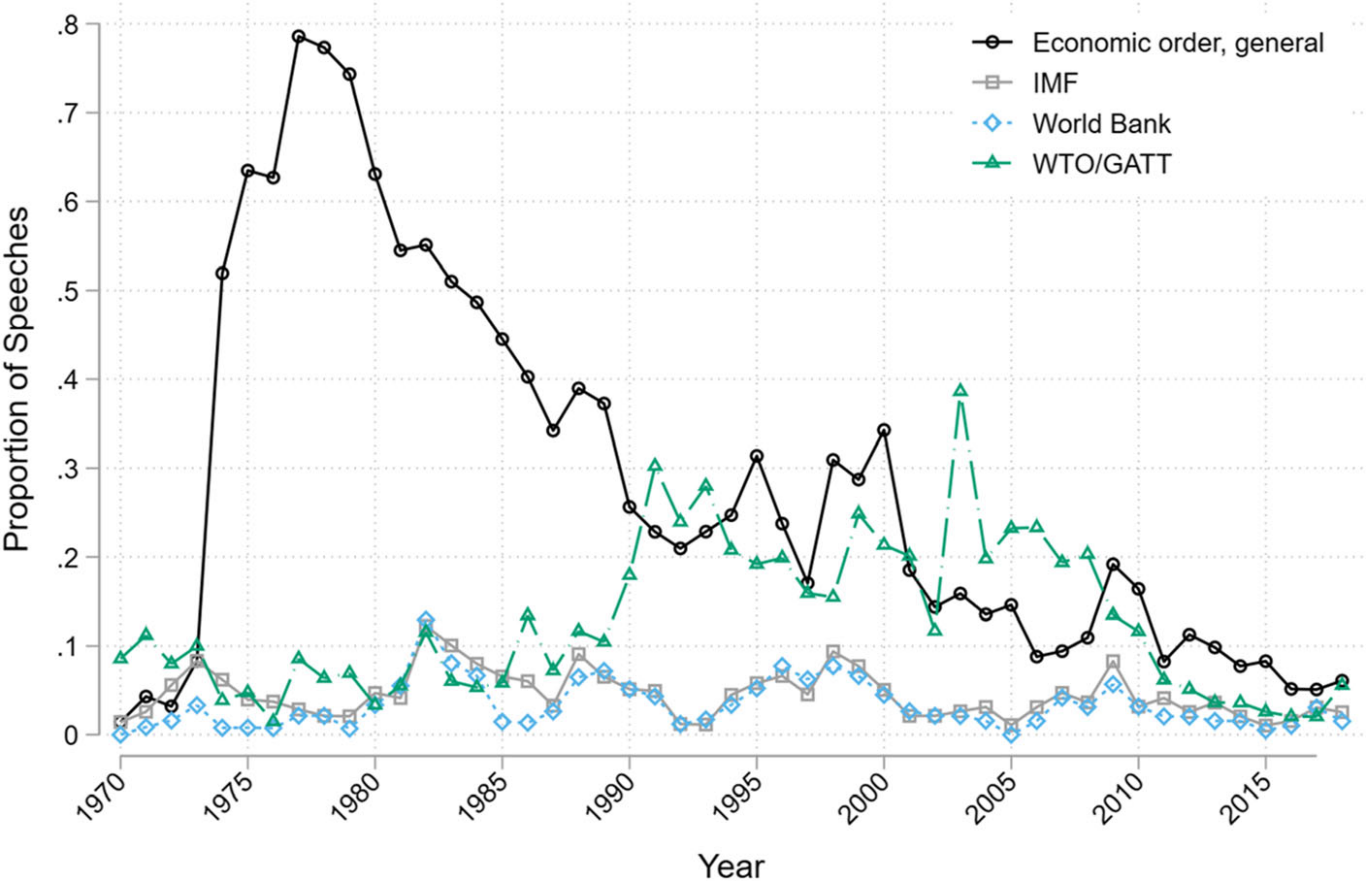
Voice against the liberal economic order and its institutions in UNGA General Debate speeches

Figure 4 plots trends in the use of voice against the liberal economic order and its key organizations. In the 1970s and 1980s, most critiques focused on the order more generally, rather than through specific mentions of some organization, and many of these critiques pertained to the NIEO agenda. The end of the Cold War did not end concerns among developing countries about dependency and the distribution of wealth and technology. Yet, these concerns no longer translated into broad-based requests to reform the order, notwithstanding temporary increases in criticisms around the time of the East Asian Crisis (late-1990s) and the Global Financial Crisis (late-2000s).

Voice about the GATT/WTO increased during the creation of the WTO system and the launch/completion of subsequent rounds of negotiations, and it has grown mostly silent after 2010. For example, we observe a notable increase in voice on multilateral trade governance in the run-up to the conclusion of the Uruguay Round in 1994. Similarly, the peak in leaders’ critical statements was in the immediate aftermath of the collapse of Doha Round trade negotiations at the WTO Ministerial Conference in Cancun in September 2003, just ten days prior to the General Debate.

**Fig. 5:**
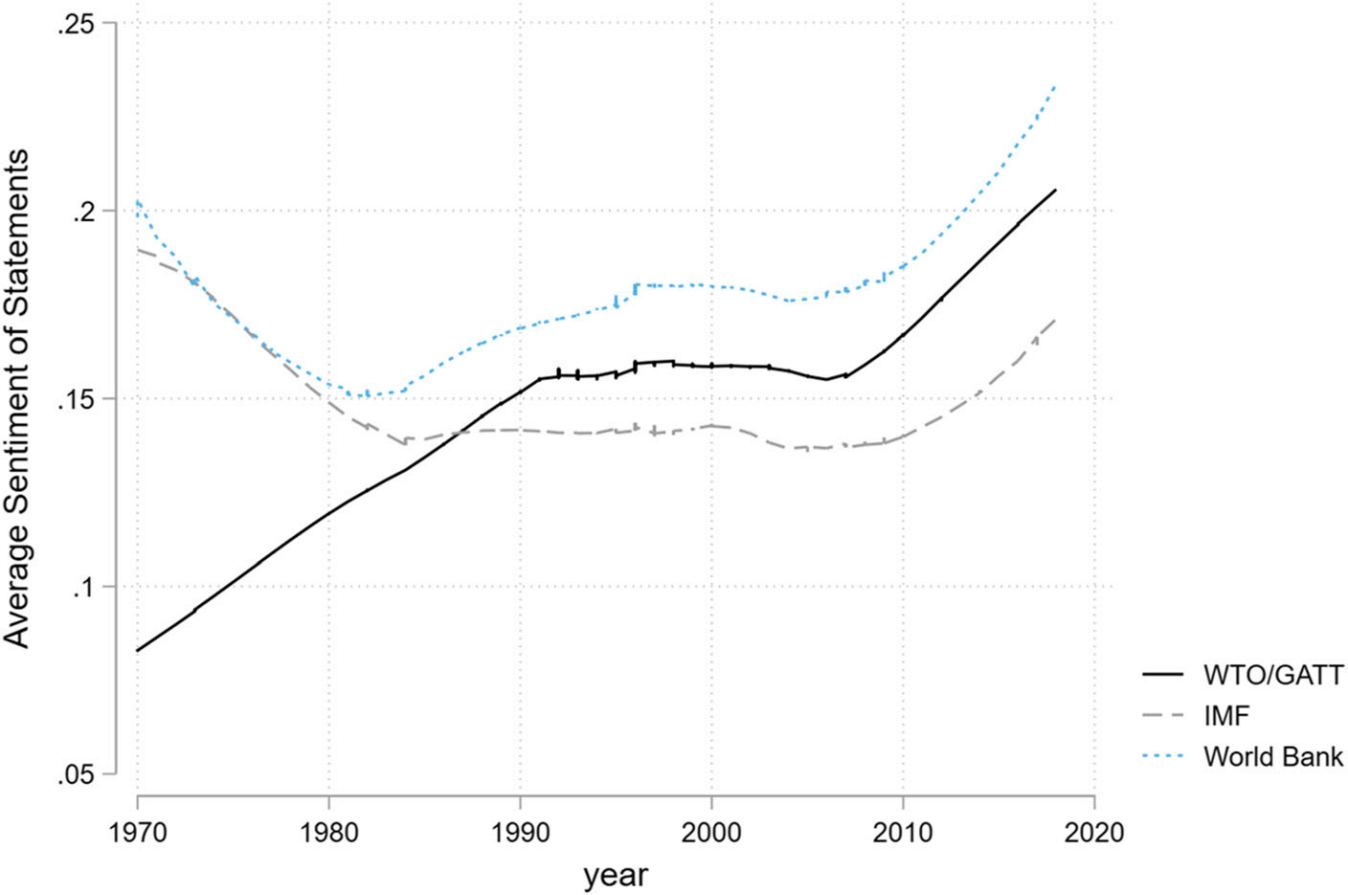
Lowess curves of sentiments expressed in statements towards global economic institutions

Figure 5 plots the average sentiments of all statements that mention specific organizations, regardless of the code we assigned to them. The global trading system originally attracted the most negative statements, which reflects that the fact that much of the NIEO battle was over issues of trade. However, by the 1990s, the IMF attracted the most negative sentiment as the organization started to roll out controversial structural adjustment programs and financial bailouts. The World Bank, by contrast, has received more positive mentions. Average sentiments towards all three organizations was flat during the first fifteen years of the post-Cold War period but has become more positive since.

Some of the post-Cold War statements with the strongest negative sentiment illustrate the insider critiques. The most negative statement (according to the automated sentiment analysis) about the WTO came from Indonesia in 1997, arguing that the WTO did not promote free trade enough:

‘In this era of trade liberalization, and in spite of the presence of the World Trade Organization (WTO), the developing countries are finding their comparative advantage rendered meaningless by an array of non-tariff barriers, preference-erosion and the misuse of anti-dumping measures and countervailing duties. Moreover, the persistent attempts of developed countries to link international trade issues with extraneous issues, such as labour standards, amount to a new form of protectionism.’

‘In this era of trade liberalization, and in spite of the presence of the World Trade Organization (WTO), the developing countries are finding their comparative advantage rendered meaningless by an array of non-tariff barriers, preference-erosion and the By contrast, the most negative statements about the global trading system during the Cold War came from Bangladesh (1979) and Burundi (1975), which stressed much more systematic challenges at the system, recalling the New International Economic Order.

Similarly, the most negative post-Cold War statement about the IMF related to concerns expressed by Argentina: ‘In 2002, the International Monetary Fund (IMF) made a serious mistake in diagnosing the problem that led to major prognostic errors and poor policy recommendations.’ In contrast, the most negative comment about the IMF or World Bank during the Cold War, from Pakistan in 1985, demanded ‘structural changes required to achieve rationality and equity in international economic relations, particularly in the international monetary and trade systems.’

**Fig. 6:**
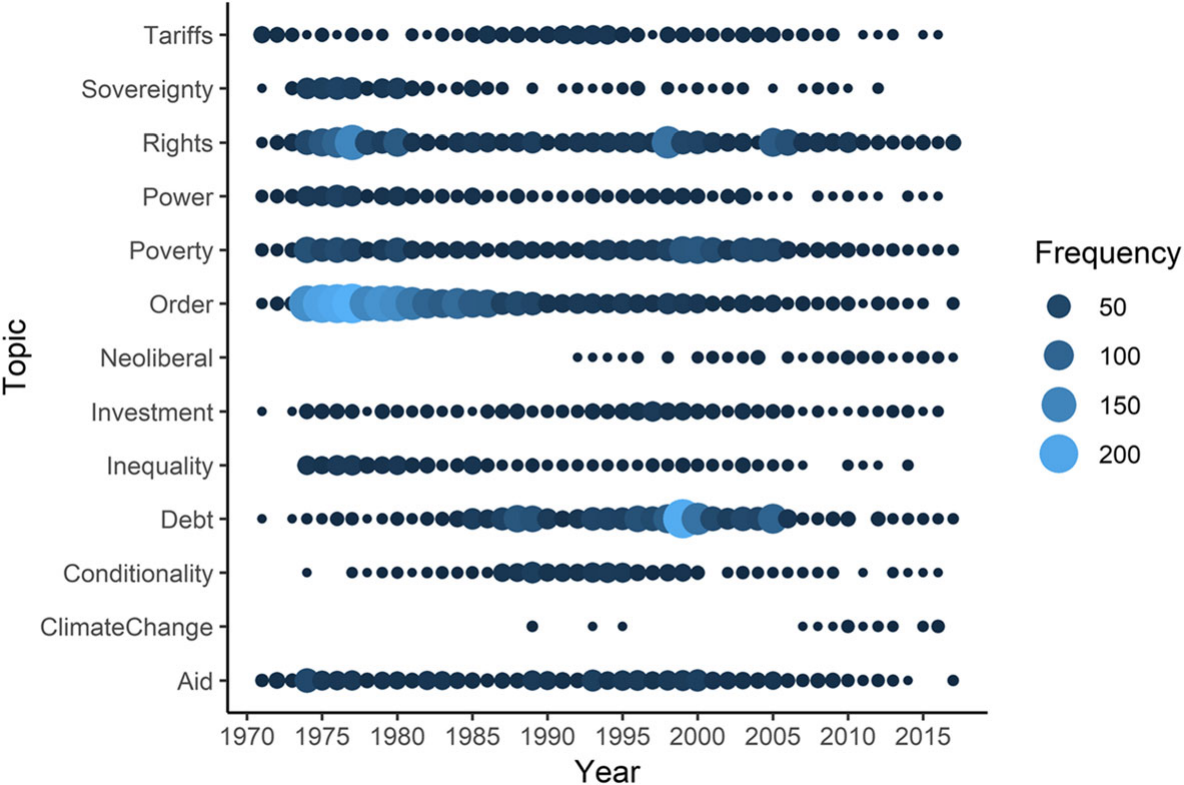
Change and continuity in the content of critiques of the liberal economic order and its institutions. **Note:** While there is no overlap in the terms that each topic captures, the topics are not necessarily mutually exclusive (e.g., mentions of “conditionality” may also reflect underlying sentiments towards “neoliberalism” even though that term may not be used by the leader). Consequently, the topics aid comparisons across time, but not necessarily between topics

Figure 6 examines the change in the kinds of criticisms more systematically by plotting the frequency of terms that appear in critical statements of international economic institutions. We used a dictionary-based approach that groups terms that relate to a concept; for example, the category conditionality includes occurrences of the terms ‘conditionality,’ ‘conditionalities,’ ‘structural adjustment,’ and ‘austerity.’ The category neoliberalism contains references to variations of the term ‘neoliberalism,’ as well as related terms, like ‘Washington Consensus.’^6^ We report the full dictionary in the appendix. These categories were selected inductively, informed by our own reading and coding of criticisms.

The figure illustrates that the mid-1970s and early 1980s were the heydays of broad-based structural critiques on the liberal order. By contrast, since then the critiques have revolved around more specific issues such as conditionality, debt, and—more recently—climate issues. Moreover, rights language has also become more pronounced in the 1990s, although not as much as in the 1970s when socio-economic rights were prominent on the UN’s agenda. Very few speeches contained language referring to neoliberalism or the ‘Washington Consensus’ even if these terms were frequently used by non-governmental activists. This suggests that few government leaders issue fundamental ideological or structural critiques on global economic institutions in the 1990s and especially the 2000s compared to earlies eras. Instead, the critiques concern the specific practices and policies of these institutions.

## Whose voice?

**Fig. 7:**
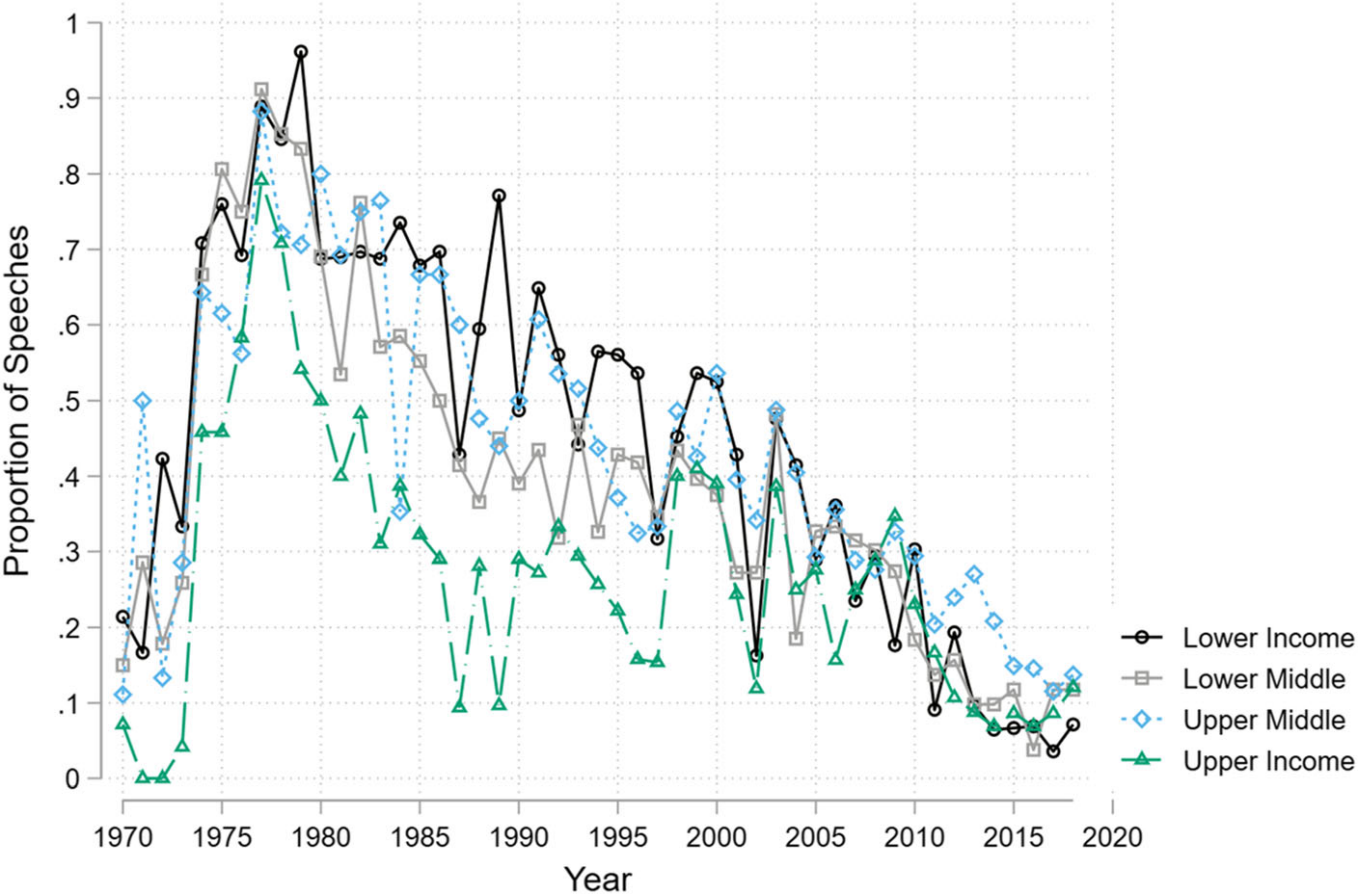
Income levels and voice against the liberal international order and its institutions

The overall trends mask differences in the characteristics of governments that do or do not voice concerns over economic institutions. The liberal economic order is often seen as structurally biased towards rich Western states (Ikenberry 2018; Kentikelenis and Babb 2019; Kohli 2020; Wade 2013). Figure 7 plots the annual proportions of voice against the liberal international order and its organizations, disaggregated by World Bank income category. Historically, lower income countries have been the most likely to use their speeches to criticize liberal international institutions. Yet, this is no longer the case. In the 1990s, 49% of speeches from low-income countries criticized international economic institutions. In the 2000s, this was 33%, and in the 2010s just 12%. By 2018, leaders from low income countries were the least likely to voice criticism at international economic institutions.

The critical voices from upper-income countries during the 1970s came primarily in the form of rhetorical support for Southern challenges to the system. For example, in 1976 Italian foreign minister Arnaldo Forlani stated that:

‘Italy is convinced of the need […] to achieve a new international economic order which will allow every nation to follow the path of development most appropriate to its own requirements and traditions and to enjoy a fair share of the world process of the production and distribution of goods. This objective will be attained only in an economic system in which the basic problems of raw materials, trade, the indebtedness of the developing countries and the transfer of technology have been solved.’

In more recent years, critiques from upper-income and upper-middle income countries appear to rise during crises, such as the Asian financial crisis in the late-1990s and the global financial crisis after 2008. For example, in 2009 French President Nicolas Sarkozy noted that:

‘The missions of both the Fund and the Bank need to be redefined. To maintain the Fund as the guardian of an orthodoxy that has been so severely shaken by the crisis would be a tragic mistake. The international system has to be reformed.’

And Greek prime-minister Alexis Tsipras explained in 2015 that ‘the neoliberal recipe we and other European countries were called to implement came at a devastating social cost and contributed to deepening the economic and fiscal crisis rather than curing it.’

## Economic openness and sentiment towards the liberal economic order

Our core argument is that criticisms to the global economic order increasingly come from states that have already adjusted their economic policies to those promoted by global economic institutions, regardless of whether they did so voluntarily or at the encouragement of these institutions. In this section, we examine this claim more systematically with a regression model with fixed effects for countries and years, thus estimating within country variation over time.

Our main expectation is that countries that are becoming more open economically were less likely to be critical of global economic institutions during the Cold War but that this relationship has reversed during the post-Cold War period. The dependent variable is a scale variable that has the value 2 if there was an endorsement, 1 for a statement of cooperation, 0 if no sentiment, −1 for voice as challenge or reform, and − 2 for a statement of exit vis-à-vis the liberal economic order and its organizational manifestations.^7^ We also tested separate models for just negative statements and just positive statements (details are in the appendix). All models include fixed effects for countries and years. This assures that the coefficients are not driven by stable between-country variation nor by specific UN sessions. For example, some UN sessions discuss economic issues, like the Millennium Development Goals, and may thus attract more commentary on economic institutions.

We employ two measures of countries’ economic policy environments. The Chinn-Ito index measures a country’s degree of capital account openness (Chinn and Ito 2006). The index captures restrictions on cross-border financial transactions as identified by the IMF’s *Annual Report on Exchange Arrangements and Exchange Restrictions,* so it directly responds to policy measures deemed important by the IMF. Trade-openness is measured by KOF’s trade globalization index, which measures both de facto and de jure trade liberalization (originally developed in Dreher (2006) and last updated in Gygli et al. (2019)). The trade globalization index is correlated at .5 with the Chinn-Ito index, reflecting that there is some variation in the extent to which countries liberalize financially and in trade. Table A3.8 in the appendix shows that both measures, on average, have increased considerably since the end of the Cold War, although less so in the 2010s, when they remained at stable levels.

We also include a measure that counts how many of the core IGOs (World Bank, IMF, GATT/WTO) a state is a party to. This captures that critiques can come from both insiders and outsiders to the liberal international economic order.

We include data on the political environments of speakers’ countries of origin. The regression models control for democracy, which is perhaps the most frequently mentioned correlate of support for global economic institutions (Mansfield et al. 2002; Milner and Kubota 2005). We use the VDEM liberal democracy scale as it is available until 2018 (Lindberg et al. 2014). Further, aside from democratic institutions, government ideology may play a major role. International economic institutions advance pro-market policies that sometimes conflict with the priorities of left-wing governments (Woods 2006). We include an indicator for whether an executive belongs to a left-wing and a right-wing party (the reference category is neither) from the Database of Political Institutions (Scartascini et al. 2018). Further, we use data by Laeven and Valencia (2018) to identify a systemic banking crisis. As a potential confounder, we include GDP per capita, which correlates with many of our independent variables but may also relate to rhetorical challenges to the order.

**Fig. 8:**
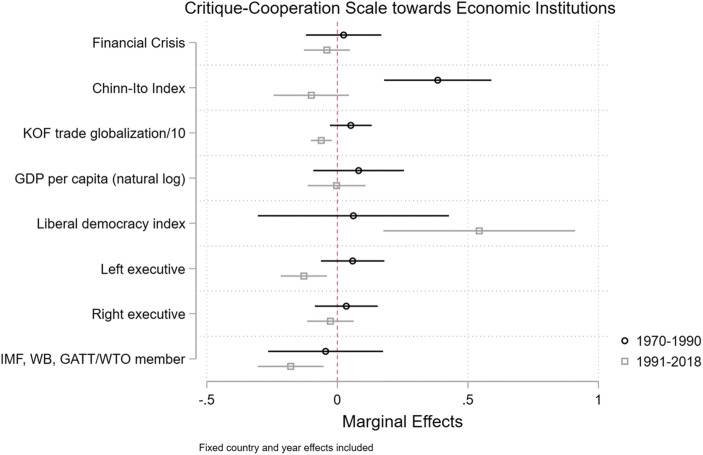
Critique-cooperation scale towards the liberal international order and its organizations (fixed effects regression model)

Figure 8 presents the findings (full tables are in the Appendix). The clearest finding is that financial openness (the Chinn-Ito Index) is correlated with more positive sentiment towards global economic institutions during the Cold War and with more negative sentiment in the post-Cold War period, although the latter coefficient is not significantly different from zero at conventional levels. Moreover, in the post-Cold War period, increased trade globalization is significantly correlated with more negative sentiment towards global economic institutions. Finally, in the post-Cold War period, countries that become members of more organizations become more critical of the global economic organizations. All of these findings are consistent with our broader argument that contestation over global economic institutions has moved towards insider contestation: countries become more likely to issue voice as they are members of the institutions and as they have already opened economically.

We do not find robust evidence that countries that experienced a financial crisis are more critical towards global economic institutions. After 1990, higher levels of liberal democracy are correlated with more positive expressed sentiment towards global economic institutions. Moreover, left-wing executives are somewhat more likely to criticize institutions than are right-wing executives, although the effects are small. All of these findings are robust to the inclusion of a measure of foreign policy ideology: a country’s ideal point estimated from UN votes (Bailey et al. 2017).^8^

Note that these are descriptive rather than causal findings. We offer a systematic analysis of the characteristics of countries that criticize global economic institutions more and less. Nonetheless, the regression analysis together with the other descriptive analyses do paint a consistent picture of patterns of rhetorical contestation over the global order. We next turn to an analysis of whether the statements we have been analyzing so far are indeed meaningful indicators of government positions towards the liberal economic order.

## Are leaders’ statements meaningful? Evidence from global debt relief debates

Studying the trends and contents of criticism of the liberal world order offers important information about the salience and valence of different institutions to world leaders, but is not able to answer the related question of how meaningful these statements are. In other words, do these statements convey accurate information about the underlying preferences that shape government policies, and—by extension—are these preferences followed through in global policy settings beyond the General Debate podium. Is voice by a leader on the UNGA linked to the behavior of state officials in other relevant fora? Are criticisms followed up, or do state representatives pursue agendas that contradict or bypass the proclaimed preferences of their leaders? To examine these questions, we focus on erstwhile controversial debates around debt relief to heavily-indebted countries: in the late 1990s, the question of debt relief became the focus of multilateral negotiations that culminated in the establishment of the Heavily Indebted Poor Countries Initiative (HIPC), to be administered by the IMF and the World Bank.

We traced statements on debt relief by state officials over the 1995–1999 period in three international settings. First, we identified debt relief-related topics statements by searching leaders’ statements at the UNGA for the keywords ‘indebted,’ ‘HIPC,’ and ‘debt relief.’ Second, we examined if and how such remarks were followed-up by ministers of finance or central bank governors at the IMF and World Bank Annual Meetings, a paramount forum for global economic governance. Like the General Debate, the Annual Meetings are held in end-September or October (i.e., after the UNGA General Debate) and offer a formal setting for concise statements by the top economic officials of many countries. However, unlike the General Debate, these Meetings attract less media attention (often limited to the financial and business press), compared to wide coverage of leaders’ statements at the UNGA. In the period covered here, approximately 60–70 such speeches were delivered each year; low-income country officials often did not attend. We accessed these statements through the World Bank’s website.^9^ Finally, we examined whether remarks at the Annual Meetings were elaborated on in the closed-door setting of the IMF’s Executive Board, where state representatives negotiate and decide on key issues in global economic governance. The Board is composed of 24 country officials who represent the entire membership of the organization. We collected the transcripts of all HIPC-related discussions from the IMF Archives.^10^

Our search for debt-related statements yielded 99 excerpts from leaders’ speeches at the UNGA, and 83 of them contained explicit critiques or calls for reform to the global debt management system (like calls for more generous or faster debt relief). Overall, we found that the statements by leaders were followed-up by their ministers of finance. As attendance at the Annual Meetings is lower than the UNGA setting, we traced 17 instances where *both* a leader voiced a call for reform of global debt management at the UNGA *and* the same country’s minister of finance or central banker attended the Annual Meetings. Of these 17 cases, 12 statements repeated or elaborated on the leaders’ UNGA remarks. These policy preferences were subsequently conveyed by country representatives at the IMF Executive Board during deliberations on the HIPC initiative.^11^

For example, early on in the debates around debt relief, Malaysia made passionate pleas for easing the debt burdens of poor countries and launched trenchant criticisms of international financial institutions. In 1995, prime minister Mahathir Mohamad noted that the IMF and World Bank had ‘merely become the instruments of power perpetuation [by rich countries, while] the debt millstone weighs heavily on the poor.’ Expanding on this theme in 1996, he told the UNGA that:

‘Despite their specific mandates to facilitate development and regulate the international monetary system, [the IMF and World Bank] are used to discipline third-world countries and to act as debt collectors for the rich North. […] The majority of poor developing countries are saddled with unsustainable levels of debt that preclude them from a share of world prosperity and growth. Debt servicing on current scales is untenable and debtor countries, as a consequence, can do little to alleviate their poverty and misery. The chilling numbers speak for themselves - more is spent on servicing debt than on financing basic programmes for health care, education and humanitarian relief.’

In the same year, these arguments were repeated by minister of finance Anwar bin Ibrahim, who told his counterparts at the IMF and World Bank Annual Meetings that:

‘despite continuous economic adjustment efforts, growth in heavily indebted poor countries (HIPCs) is still retarded. This is because HIPCs are diverting resources toward debt repayment, making it almost impossible to expend capital on infrastructure and education. Experiences in many countries have proven that external assistance to relieve repayments was crucial for the undivided pursuit of economic policies to promote growth. We are relieved that the IMF has decided on the modalities to finance the Enhanced Structural Adjustment Facility (ESAF). Although the understanding reached is less than desired, it nevertheless will enable the Fund to participate in the HIPC initiative. […] The Fund must expedite efforts to ensure adequate resources for a self-sustaining ESAF.’

Similar concerns regarding the debt situation were also expressed by the Indonesian foreign minister Ali Alatas, who explained at the UNGA that:

‘Indonesia has long advocated a set of principles for managing the debt problem, calling for a ‘once-and-for-all’ settlement of the debt problems of developing countries, including multilateral debts, as well as the cancellation of the debts of the most severely affected, low-income developing countries. In this context, we welcome and support the joint proposal of the World Bank and the [IMF], which offers effective alternatives for reducing the overall debt burdens of heavily indebted poor countries to sustainable levels. While this initiative could be further refined, it is Indonesia’s fervent hope that, at the forthcoming meetings of the World Bank and the IMF, this proposal will finally receive the support that it needs and deserves from the developed countries.’

Further supporting this policy agenda, minister of finance Mar’ie Muhammad clarified at the IMF and World Bank Annual Meetings that:

‘While Indonesia supports the heavily indebted poor countries debt initiative, we would note that there are elements of the proposal that need strengthening. First, Indonesia hopes that the Bank and the Fund can be more forthcoming in their contributions. […] Second, while Indonesia appreciates the need to monitor economic reforms for some time to ensure that they are well founded and effectively implemented, we would hope that the decision point under the initiative could be shortened.’

This imperative to speed-up the process of access to debt relief was also expressed by J.E. Ismael, the country’s representative to the IMF, during a Board meeting:

‘The HIPCs will get full benefits from the proposed plan only after six years, which is quite a long time for such distressed countries. The need for a more adequate debt treatment of HIPCs has been discussed for many years. […] It seems that the implementation phase of the proposed plan could be shortened for the countries that have good records with the World Bank and the Fund.’

We found few statements on the global debt regime by high-income country officials, and their policy preferences conveyed across the three fora were consistent. For instance, French prime minister Lionel Jospin and central bank governor Jean-Claude Trichet used nearly-identical language in 1999 to convey the French position vis-à-vis debt relief. Leaders from Britain, Norway and Sweden stand out for making reference to heavily-indebted poor countries in more than two UNGA speeches over the five-year period covered here. For example, Britain—the only ‘Group of 7’ country to make reference to debt issues—defended debt relief and even called for more ambitious implementation targets. In 1998, prime minister Tony Blair told the UNGA:

‘[we] have to ease the debt burden on the poorest countries. Britain has proposed the Mauritius Mandate to speed up assistance for those in the debt trap who are genuinely ready to help themselves out of it. By the year 2000 all qualifying highly indebted countries should have embarked on a systematic process of debt reduction, with the aim of a permanent exit from their debt problems. But we need to make sure it happens.’

Following up on this statement at the Annual Meetings a few days later, UK chancellor of the exchequer, Gordon Brown, noted that:

‘Vigilance in national economic policies today must be matched by a willingness to reform the international financial system to secure greater stability tomorrow. As British Prime Minister Tony Blair said in New York two weeks ago, we must create a new global framework which will have to mirror, at a global level, national regimes for transparency, supervision, crisis management, and stability. […] I am pleased that this week, we have agreed on new procedures for advancing debt reduction to post-conflict countries. And to meet our Mauritius Mandate targets, we need to aim for 22 countries to reach the decision point in the HIPC process by the end of 1999. […] All countries must stand ready to provide the resources to make progress by 2000.’

In December 1998, Stephen Pickford, UK representative to the IMF, repeated Gordon Brown’s call for increasing the resources available for HIPC. Pickford reminded the IMF Executive Board that the financing of the HIPC initiative had ‘a long and tortuous history,’ and stressed that ‘we cannot continue to make commitments to countries and other creditors on the provision of debt relief without the resources to back those commitments.’ As a way forward, the UK representative elaborated that his authorities supported selling part of the IMF gold reserves and increasing their bilateral contributions.

In sum, employing this comparison of discourse in three global governance arenas, we demonstrate that leaders’ statements at the UNGA convey meaningful information on underlying state preferences. We showed consistency across three different global fora, which suggests a degree of continuity in articulated policy positions. The fact that country representatives “recycle” some of the same material strengthens our argument that leaders’ speeches are not just performative but followed through by other country officials. The main difference between these international fora appears to be the mode of delivery of remarks, rather than the content of the policy preferences. UNGA speeches relied on vivid and passionate language to deliver the main message, while statements at the two global economic governance arenas—the IMF and World Bank annual meetings, and the IMF Executive Board—were more temperate. In the case of the IMF Board, remarks were of a technical nature, and lacked the affective dimension of political speeches in the two other venues. In other words, states’ policy preferences became more conforming to the technocratic, consensus-based nature of deliberations in global economic governance, where the audience is primarily other economic officials, international bureaucrats and the business press, compared to the more animated speeches by leaders at the UNGA.

## Conclusions

World leaders spend less and less time on the podium of the UNGA criticizing the liberal international order. Notwithstanding current narratives about its fragility, a declining number of leaders use their high-profile UN speeches to challenge global economic institutions or the order. Indeed, a defining feature of recent years is the relative silence about the order and its institutions. Silence—while notoriously difficult to interpret—can be seen as a passive granting of legitimacy: given the rarity of exit, de-legitimation through discourse is a key avenue available to challenger states but very few leaders take this route.

Why is criticism of the liberal world order in the UNGA at an all-time low? While our findings do not present a comprehensive answer to this question, our analysis points to three directions: country preferences, broader geopolitical shifts, and institutional inertia. As far as country preferences are concerned, leaders might no longer expend time criticizing the liberal international order for many reasons. For some countries this order has delivered concrete benefits, and some political leaders have ideological preferences aligned with its policy prescriptions. But even skeptics might have diminishing incentives to criticize: while the present order might not suit their interests, they prefer it to an alternative—and likely—order that is based primarily on bilateral deals and a more naked exercise of power, as the U.S. is currently attempting. The absence of a concrete set of policy alternatives that commands the support—in rhetoric and in practice—of enough governments to become a serious challenger ends up inadvertently boosting the legitimacy of present arrangements, even if contingently and provisionally so. We return to this issue below.

Broad geopolitical shifts also have a role to play. Although there are still some vocal attempts to delegitimize the liberal institutional order from socialist governments, especially in Latin America, most challenges now stem from countries that are wealthier and better integrated into the global economy. We suggest that this is a reflection of an underlying change in the nature of contestation towards global economic institutions: the Cold War era of insider-outsider conflict has given way to insider contestation over the rules of the game. Membership in the core global economic institutions is now near universal, encouraging contestation over rules and practices rather than the order itself.

Finally, the liberal economic order has been comprehensively institutionalized, so that it is subject to strong inertial forces that increase its perceived immutability and staying power. After decades of global economic governance organizations diffusing free market policies around the world (Simmons et al. 2008), many countries have comprehensively implemented the main tenets of this order and take it for granted. This taken-for-granted quality has become an attribute of the liberal institutional order, and can explain why it is seen by many leaders as a topic that has been settled and no longer requiring debate.

Of course, even though criticism of the liberal world order on the UNGA is low, it matters a great deal that the order’s main protagonist, the United States, is now among its fiercest critics. For example, in his 2019 address to the UN, Donald Trump posed fundamental challenges to the WTO and the order it underpinned:

‘The World Trade Organization needs drastic change. The second-largest economy in the world should not be permitted to declare itself a “developing country” in order to game the system at others’ expense. For years, these abuses were tolerated, ignored, or even encouraged. Globalism exerted a religious pull over past leaders, causing them to ignore their own national interests’ (White House 2019).

This type of direct criticism by a leader of the U.S.—the creator and erstwhile guarantor of the liberal economic order—breaks with historical patterns. Whether this kind of reorientation of US international economic policy is permanent or temporary is a topic of sustained speculation, but it has already had tangible consequences in the functioning of international institutions. The global pandemic has thrown these consequences into sharp relief. For instance, the U.S. administration blocked IMF plans to increase international liquidity to combat the economic fallout of Covid-19 (Tooze 2020).

Although much of international politics is about influencing international and domestic audiences through speech, our understanding of just how this works still has some way to go. We sketch the outline of a broader research agenda for the study of legitimation processes vis-à-vis the international order.

First, rhetoric in the UNGA is only the tip of the iceberg in efforts to legitimize or delegitimize an institutional order. Countries have a wide gamut of options to follow up leader rhetoric: from outright withdrawal (Eilstrup-Sangiovanni 2018; von Borzyskowski and Vabulas 2019), to attempts to reorient the activities of institutions by working through their governance channels (Kentikelenis and Babb 2019; Kentikelenis and Seabrooke 2017), or to setting up alternative structures (Kring and Gallagher 2019; Lipscy 2015b, 2016). The temporality and interlinkages between these processes is insufficiently understood: What course do leaders choose to grant or withhold legitimacy from global institutional arrangements, and under what set of circumstances? How effective are these discursive attempts, and what determines their likelihood of success?

Future research can examine the extent to which the proclaimed policy priorities of leaders are indeed followed through by country officials in other international fora. We offered a glimpse of this in our comparison of the highly public UN setting and less public IMF and World Bank meetings. However, consistency in country officials’ statements across different global fora—even in closed-door settings—does not capture whether these are followed through, which is the outcome not only of states’ policy preferences but also inter-state bargaining and compromise. Subsequent work can link leaders’ discourse to state activities like positioning in international negotiations, participation in programs run by international institutions, compliance with commitments or voting patterns at the UN and other international fora.

Relatedly, solely examining government rhetoric and actions to evaluate the legitimacy of the international economic order also has blind spots: civil society also has a key role in this process (Nielson et al. 2019; Rauh and Zürn 2019). For instance, it was major worldwide mobilization that resulted in the cancellation of the WTO meeting in Seattle in 1999, events that—in the words of former GATT Director-General Peter Sutherland—revealed ‘a fundamental deficit in effective political support for the WTO system’ (cited in Slobodian 2018, p. 275). Global civil society mobilization can offer alternative narratives and frames for world leaders and diplomats to draw on—understanding when and how these alternative visions to the world order make their way into the UN system is a task for future studies.

Second, leader discourse in the UNGA is not only directed towards a global audience, but also has domestic recipients: it is possible that many remarks are included for consumption within the leader’s home country. Recent work has highlighted politicians’ recourse to domestic media to support or criticize international organizations (Schmidtke 2019), and—given their domestic visibility—UNGA statements are part of this process. What implications do leader narratives about the global order have for its perceived legitimacy among different constituencies or target audiences? For example, extensive and favorable domestic media coverage of critical speeches can influence national public opinion, thereby impacting the policy outlook and foreign policy ideologies of future governments. More broadly, UNGA speeches provide an avenue to further examine the interactions between domestic and international speech at a time when international institutions are becoming increasingly salient in domestic politics (de Vries and Hobolt 2020).

Third, when criticism or support of the liberal order or an institution is shared by a sufficient number of leaders, this may influence the legitimacy of attempts at further international integration (Tallberg and Zürn 2019). UNGA speeches can be a source for further understanding alliances or similarities between sentiment or preferences of different countries: do leaders reference each other, express similar priorities, target the same institutions, or set forth a common vision? And what does all this depend on—regional characteristics, political orientation, economic links, historical ties? Such evidence could yield indicators that complement established measures of voting alignment at the UNGA (Bailey et al. 2017).

Fourth, an important feature of the General Debate corpus is that it allows us to analyze silence as well as speech. Because (almost) each UN member-state gives a speech each year, we can study if and when they decide to use their scarce time to criticize or endorse global economic institutions. Such analysis can go beyond what we offered in this article. For example, we found no evidence that leaders motivate joining new institutions, such as the AIIB, by critiquing or threatening to exit existing ones. This does not mean that such institutions do not effectively function as exit options that can incentivize reform (Lipscy 2016). Rhetoric is not all that matters but it is nonetheless interesting that leaders do not explicitly delegitimize existing institutions even as they contemplate alternatives. This deserves further scholarly attention.

Finally, advances in automated content analysis could be exploited to extend the study of legitimacy of the global order beyond the corpus of UN General Debates. We only scratched the surface of the possibilities of automated text analysis in our application of sentiment analysis. For example, our human coding can be used to develop machine learning algorithms to detect criticisms against international institutions in a corpus of international or national speeches. Such an application of machine learning could help us better understand, for instance, the extent to which contestation over global economic institutions features in the domestic politics of countries.

At the time of writing, a global pandemic has exacted a devastating human toll and resulted in deep economic crises, and climate change is ever-more-widely recognized as an existential threat to humanity. The liberal international order has not been able to effectively respond to these crises: the support of multilateral economic organizations has been far below estimated need (Stubbs et al. 2021) and governance constraints prevent forceful action (Biermann 2014). In light of these failures, unrest is brooding and legitimacy challenges—whether by politicians, civil society or business interests—are intensifying (Colgan et al. 2020; Hale 2020), even though they have not yet become prominent on the UNGA podium. For example, calls for a Global Green New Deal propose a radical rethinking of the institutional arrangements of globalization, comparable to the radical overhauls promoted by the ultimately unsuccessful New International Economic Order agenda in the 1970s and 1980s. It is possible that the decline in criticisms towards the liberal world order that we documented only reflects the calm before the approaching storm.

## Electronic supplementary material

ESM 1 (R 3 kb)

ESM 2 (CSV 19019 kb)

ESM 3 (DTA 3911 kb)

ESM 4 (DTA 28 kb)

ESM 5 (PDF 471 kb)

ESM 6 (DO 12 kb)

ESM 7 (DTA 17721 kb)

ESM 8 (DTA 35068 kb)

ESM 9 (CSV 1181 kb)

## Footnotes

1 A limitation of this approach is that our analysis might not pick up some critiques that do not explicitly mention institutions, but are more diffuse.

2 Data on withdrawals from international organizations is available from von Borzyskowski and Vabulas (2019).

3 Data on IMF programs by Dreher and Gassebner (2012).

4 We did not code these statements as exit because they typically talked about reform rather than leaving institutions, although some at least suggested replacing institutions.

5 We chose this representation for a number of reasons. Polynomial smoothers did not capture the trend accurately whereas annual fluctuations are somewhat noisy. The differences between the annual averages of the critical and cooperative statements are statistically significant (at the 5% level) in all years other than 1970–1975.

6 We also estimated a topic model (using Latent Dirichlet Allocation) but that did not return topics with some terms we deemed substantively important, such as neoliberalism. The results from the topic model were more difficult to interpret but generate a similar substantive insight: that general structural critiques are down and more specific critiques have increased.

7 If a statement of voice was followed by a statement of cooperation, we still counted it as voice. Often these are instances where a leader criticizes an international organization for a specific set of actions but then expresses broader support, which together fits our definition of voice. 52% of all observations have a negative value and 40% are 0 in the Cold War period compared to 28% and 60% in the post-Cold War period.

8 See more detailed findings in the appendix. We excluded this measure from the main specification for concern that UN vote-based measures and data from UN speeches could reflect similar data generation processes.

9 Available from http://documents.worldbank.org/curated/en/docsearch?query=Boards%20of%20Governors%20summary%20proceedings (accessed October 22, 2018).

10 Available from http://archivescatalog.imf.org/ (accessed October 22, 2018).

11 Here, we are exclusively interested in whether leader’s statements are followed up by other country officials. Of course, there was a higher overall number of references to debt management at the IMF and World Bank Annual Meetings, as that is the key venue where relevant decisions are being made.
