# Supplementary material for: Legitimacy challenges to the liberal world order: Evidence from United Nations speeches, 1970–2018
Source: Rev Int Organ. 2020 Oct 9;16(4):721–54. doi: 10.1007/s11558-020-09404-y (PMC7546938; doi:10.1007/s11558-020-09404-y)

Alexander Kentikelenis (alexander.kentikelenis@unibocconi.it)
Department of Social and Political Sciences, Bocconi University

Erik Voeten (ev42@georgetown.edu)
Edmund E. Walsh School of Foreign Service & Government Department, Georgetown University

Legitimacy Challenges to the Liberal World Order: Evidence from United Nations Speeches, 1970-2018

—Web Appendix—

1. Codebook *2*
Approach 2
Dictionary 2
Coding practices 3
Examples provided to coders 3
Coding Process and Reliability 5
2. Dictionary for Figure 5 .... *7*
3. Regression Analyses *8*
A3.1. Table underpinning Figure 8 8
A3.1.1. Correlation matrix 8
A3.1.2. Descriptive statistics 9
A3.2. Table including UN Ideal Point 9
A3.3. Table including interaction between openness and Chito-Inn 10
A3.4. Lagged dependent variable (random country effects) 11
A3.5. Separate Regressions for Criticism and Cooperation 12
A3.6. Table including IMF/World Bank program participation 13
A3.7. Changes in average financial and trade openness over time 14

1. Codebook

Approach

We created a detailed dictionary (see below) of search terms that plausibly identify speech regarding the following general categories:

1. Liberal international economic order
2. World Bank
3. International Monetary Fund
4. World Trade Organization and General Agreement on Tariffs and Trade

We searched the corpus of UN speeches to create a spreadsheet with the frequency of terms found in each speech for each category. The spreadsheet also stored the excerpts in which the terms were mentioned. The speeches that mention our terms are then manually coded.

Dictionary

LIBERAL INTERNATIONAL ECONOMIC ORDER

"International financial institution"
"Bretton"
"free trade"
"Development bank"
"International Economic Order"
"NIEO"
"liberal order"
"neoliberal"
"structural adjustment"
"Washington Consensus"
"structural reform"
"conditionality"
"debt management"
"HIPC"
"indebted poor countries"
"Highly indebted"
"Highly-indebted"
"debt restruct"
"Algiers Conference"

IMF

"International Monetary Fund"
"IMF"
" IMF"

WORLD BANK

"World Bank"
"International Bank for Reconstruction"
"International Development Association"
"International Finance Corporation"
"IBRD"
" IDA"
" IFC"

WTO/GATT

"World Trade Organization"
"World Trade Organisation"
"WTO"
"Agreement on Tariffs"
"GATT"
"Doha Round"
"Uruguay Round"
"Doha agenda"
"Trade round"

Coding practices

We assigned each non-zero entry in the spreadsheet with speech excerpts using the following letter codes. In instances where two categories clearly and somewhat equally apply, we assigned multiple codes separated by a comma.

lo Loyalty as endorsement: Speakers express unambiguous support for or commitment to an institution. This includes calls for countries to better abide by the rules of an institution in order to make the institution work better. It also includes efforts to defend an institution, and countries expressly saying that they are working with an institution to address some issue.

cp Loyalty as cooperation and participation: Speakers make factual statements about their country's involvement in an institution without an explicit statement of support or criticism.

vr Voice as call for reform: Speakers express conditional support for an institution, and articulate constructive calls for reform without fundamentally challenging the institution. This includes calls for institutions to improve their performance and indications of a willingness to engage in discussions about reform.

vc Voice as challenge: Speakers articulate a fundamental challenge or criticism to an institution. Compared to "vr," we are looking for speeches that explicitly charge institutions (or the order) with some important deficiency and/or that call for fundamental reforms in terms of representation and/or policies.

ex Voice as intention to exit: Speakers announce a (conditional or unconditional) intention to abandon an institution, or in other ways suggest that an institution should be abandoned or that countries would be better off not partaking in the institution's activities. This includes calls for an institution to be replaced by an alternative.

na Not applicable: Speakers make a reference to one or more of the dictionary words, but they carry no particular valence and cannot be coded in one of the categories above. This includes statements that are not about international institutions or the international order but about economic or human rights policies.

fp False positive: Remarks that were picked up on the basis of dictionary words, but that are not relevant to the project's remit.

Examples provided to coders

Loyalty as endorsement: Speakers express unambiguous support for or commitment to an institution. This includes calls for countries to better abide by the rules of an institution in order to make the institution work better. It also includes efforts to defend an institution, and countries expressly saying that they are working with an institution to address some issue.

Source: NLD_28_1978.txt
Coding category: WTO/GATT and IMF

The North-South dialogue ought not to deflect attention from creeping protectionism and other negative developments which directly affect the position of developing countries. Timely corrective and preventive measures in this field are at least as important as an increased transfer of resources. Here lies

an urgent task for Governments and international organizations and their executive officers, in particular GATT, IMF, UNCTAD, OECD and the United Nations itself.

Loyalty as cooperation and participation: Speakers make factual statements about their country's involvement in an institution without an explicit statement of support or criticism.

Source: UZB_2017
Coding category: GENERAL IFIs
Cooperation with international financial institutions is being successfully developed, and partnership with the European Bank for Reconstruction and Development has been renewed. I want to note that the contents of the strategy are fully consistent with the United Nations Sustainable Development Goals.

Voice as call for reform: Speakers express conditional support for an institution, and articulate constructive calls for reform without fundamentally challenging the institution. This includes calls for institutions to improve their performance and indications of a willingness to engage in discussions about reform.

Source: ARG_32_1977.txt
Coding category: WTO/GATT
We believe that a wide-ranging and open dialogue between multinational integration groups and GATT might not only yield reciprocal benefits but also abundantly enrich the norms of that international organization.

Source: NLD_30_1976.txt
Coding category: WORLD BANK
Apart from the continuing dialog and negotiations in Paris, the first test case will be the forthcoming UNCTAD negotiations on the integrated program for commodities and the common fund. Other negotiations, such as those on multilateral trade, debt relief, monetary reform and the fifth replenishment of the International Development Association will equally require a political effort from all Members of this Organization. These are fundamental issues of equal opportunity and justice. Failure to act and agree may have serious political implications. The appeal from Colombo should not remain unheeded.

Source: VCT_2017
Coding category: LIBERAL INTERNATONAL ECONOMIC ORDER
The General Assembly and the Economic and Social Council must squarely and frontally address reforms to our international financial architecture, in particular the Bretton Woods institutions. For Caribbean States, the issue of declining correspondent banking relationships is a grave and gathering threat to our continued growth and ongoing connections to the global economy. The United Nations, the Group of 20 and international financial institutions cannot shirk this issue as a private banking matter. It is a threat to development of the highest priority, and it demands a coordinated, political solution.

Voice as challenge: Speakers articulate a fundamental challenge or criticism to an institution. Compared to "vr," we are looking for speeches that explicitly charge institutions (or the order) with some important deficiency and/or that call for fundamental reforms in terms of representation and/or policies.

Source: ARG_30_1975.txt
Coding category: LIBERAL INTERNATONAL ECONOMIC ORDER
the almost unanimous recognition by member states of the need to advance slowly but surely towards the establishment of a new international economic order allows us to hope that this process of liberation is already under way.

Source: ARG_33_1978.txt
Coding category: LIBERAL INTERNATONAL ECONOMIC ORDER
possible to conclude that the life cycle of many of the elements in that framework of relations has come to an end and that therefore it is necessary to readjust the structure of international economic relations to present-day realities. that aim is nothing other than the establishment of a new international economic order, and that entails the need to undertake progressive and profound reforms.

Not applicable: Speakers make a reference to one or more of the dictionary words, but they carry no particular valence and cannot be coded in one of the categories above. This includes statements that are not about international institutions or the international order but about economic policies.

Source: NLD_30_1977.txt
Coding category: LIBERAL INTERNATONAL ECONOMIC ORDER
I see no contradiction between the concept of growth and global interdependence and the strategy to eradicate poverty and provide for basic human needs. The development policy of the Netherlands will in fact be guided by both these concepts. We shall strive for selective growth and positive readjustment measures in developed countries, for structural changes in the world economy and, thus, for the realization of the new international economic order. At the same time, the Netherlands policy will be directed at the eradication of mass poverty and unemployment.

False positive: Remarks that were picked up on the basis of dictionary words, but that are not relevant to the project's remit.

Source: ARG_26_1971.txt
Coding category: LIBERAL INTERNATONAL ECONOMIC ORDER
On the issue of structural reform, we note here that Australia has advocated expanding the permanent membership of the Security Council to make it more compatible with geographical realities , through the addition of Japan , India , Brazil , an African country and Indonesia

Coding Process and Reliability

After extracting speech excerpts based on the dictionary reported above, we collaborated with graduate students to complete the coding. A first 'training' set of speeches (approximately 200 excerpts) was coded by the co-authors of the article to generate and fine-tune the codebook. We then assigned three coders partially overlapping samples. One of our original three coders turned out to produce coding that varied substantially from the other coders. Our own assessment was that while the quality of the first two coders was high, the third coder was not producing reliable codes. We subsequently moved forward with just the two coders and discarded all the codes from the third person. Originally, we coded two types of voice: *Voice as call for reform* articulates constructive

recommendations for changes to the system, like calls for institutions to improve their performance or indications of willingness to engage in discussions about reform. *Voice as challenge* articulates a fundamental criticism or challenge. In the analysis, we combine the two types of *Voice* as reflections of criticism. This is partially for reasons of simplicity but also because we found that while inter-coder reliability was high for most categories, coders had more difficulties differentiating voice as a call for reform or challenge. Inter-coder agreement for all four categories discussed in the body of the paper exceeds 90%.

2. Dictionary for Figure 5

Category	Terms
Aid	"aid", "assistance"
Climate Change	"climate change", "global warming"
Conditionality	"conditionality", "conditionalities", "structural adjustment", "austerity"
Debt	"debt", "indebted", "HIPC", "Baker Plan", "Brady Plan"
Inequality	"inequality", "equity", "distribution", "distributive", "inequalities"
Investment	"investment", "corporation", "corporations", "multinationals", "enterprise", "enterprises”
Neoliberal	"neoliberal", "neo-liberal", "washington consensus", "neoliberalism", "neo-liberalism"
Order	"order", "structural change", "structure"
Poverty	"poverty", "food", "poor", "hunger", "destitution"
Power	"power", "powerful", "powerless", "weak", "hegemon", "hegemony"
Rights	"right", "rights"
Sovereignty	"sovereign", "sovereignty", "interference", "intervention", "non-interference"
Tariffs	"tariff", "tariffs", "trade barrier"

3. Regression Analyses

A3.1. Table underpinning Figure 8

(Fixed country and year effects)

VARIABLES	(1)<br>ECONscale	(2)<br>ECONscale
Financial Crisis	0.02	-0.04
	(0.07)	(0.04)
Chinn-Ito Index	0.38***	-0.10
	(0.10)	(0.07)
KOF trade globalization/10	0.05	-0.06***
	(0.04)	(0.02)
GDP per capita (natural log)	0.08	-0.00
	(0.09)	(0.06)
Liberal democracy index	0.06	0.54***
	(0.19)	(0.19)
Left executive	0.06	-0.13***
	(0.06)	(0.05)
Right executive	0.03	-0.03
	(0.06)	(0.05)
IMF, WB, GATT/WTO member	-0.04	-0.18***
	(0.11)	(0.06)
Constant	-0.92	0.40
	(0.74)	(0.50)
Observations	1,976	3,409
R-squared	0.14	0.05
Number of CIDnum	113	143

A3.1.1. Correlation matrix

	ECONsc~e	fincri~s	ka_open	KOFTrGI	e_migd~n	v2x_li~m	left	right	member
ECONscale	1.0000								
fincrisis	-0.0136	1.0000							
ka_open	0.1384	0.0010	1.0000						
KOFTrGI	0.1724	-0.0084	0.5306	1.0000					
e_migdppcln	0.1562	0.0149	0.5461	0.6872	1.0000				
v2x_libdem	0.1496	0.0496	0.4200	0.4748	0.5879	1.0000			
left	-0.0705	0.0044	-0.0838	-0.0058	0.0046	0.1440	1.0000		
right	0.0765	0.0360	0.1948	0.1426	0.2389	0.3404	-0.3132	1.0000	
member	0.0102	0.0277	0.0705	0.1004	0.1271	0.3215	0.0277	0.1267	1.0000

A3.1.2. Descriptive statistics

Variable	Obs	Mean	Std. Dev.	Min	Max
ECONscale	7,853	-.2441105	.7073805	-2	2
fincrisis	7,833	.0578322	.2334409	0	1
ka_open	6,732	.4515846	.3596647	0	1
KOFTrGI	7,338	4.894468	1.811534	.9276916	9.696732
e_migdppcln	6,134	8.695606	1.213474	4.89784	12.30464
v2x_libdem	7,049	.3554028	.2820734	.0024428	.9034218
left	7,853	.2624475	.4399926	0	1
right	7,853	.1725455	.377878	0	1
member	7,638	2.526316	.7361137	0	3

A3.2. Table including UN Ideal Point

VARIABLES	(1)<br>CW	(2)<br>PCW
Financial Crisis	0.02	-0.04
	(0.07)	(0.04)
Chinn-Ito Index	0.35***	-0.11
	(0.11)	(0.07)
KOF trade globalization/10	0.04	-0.07***
	(0.04)	(0.02)
GDP per capita (natural log)	0.08	0.02
	(0.09)	(0.06)
Liberal democracy index	0.08	0.31
	(0.19)	(0.19)
Left executive	0.06	-0.12**
	(0.06)	(0.04)
Right executive	0.03	-0.04
	(0.06)	(0.05)
IMF, WB, GATT/WTO member	-0.03	-0.18***
	(0.11)	(0.06)
UN Idealpoint	0.07	0.27***
	(0.06)	(0.05)
Constant	-0.92	0.33
	(0.74)	(0.50)
Observations	1,976	3,409
R-squared	0.14	0.05
Number of CIDnum	113	143

A3.3. Table including interaction between openness and Chito-Inn

VARIABLES	(1)<br>CW	(2)<br>PCW
Financial Crisis	-0.00	-0.13
	(0.10)	(0.08)
Chinn-Ito Index	0.38***	-0.12
	(0.11)	(0.07)
Financial crisis*Chinn-Ito	0.09	0.16
	(0.26)	(0.12)
KOF trade globalization/10	0.05	-0.06***
	(0.04)	(0.02)
GDP per capita (natural log)	0.08	0.00
	(0.09)	(0.06)
Liberal democracy index	0.06	0.55***
	(0.19)	(0.19)
Left executive	0.06	-0.13***
	(0.06)	(0.05)
Right executive	0.03	-0.03
	(0.06)	(0.05)
Constant	-0.94	0.34
	(0.74)	(0.51)
Observations	1,976	3,409
R-squared	0.14	0.05
Number of CIDnum	113	143

Standard errors in parentheses
*** p<0.01, ** p<0.05, * p<0.1

A3.4. Lagged dependent variable (random country effects)

VARIABLES	(1)<br>CW	(2)<br>PCW
ECONscale = L,	0.16***	0.15***
	(0.02)	(0.02)
Financial Crisis	-0.06	-0.02
	(0.07)	(0.04)
Chinn-Ito Index	0.26***	0.00
	(0.06)	(0.04)
KOF trade globalization/10	0.00	0.02*
	(0.01)	(0.01)
GDP per capita (natural log)	0.04**	-0.00
	(0.02)	(0.01)
Liberal democracy index	0.40***	0.03
	(0.08)	(0.06)
Left executive	-0.02	-0.07**
	(0.04)	(0.03)
Right executive	0.05	0.08**
	(0.05)	(0.03)
Constant	-0.64***	0.03
	(0.20)	(0.14)
Observations	1,824	3,366
Number of CIDnum	113	143

A3.5. Separate Regressions for Criticism and Cooperation

	Cold War		Post-Cold War	
VARIABLES	Critique	Cooperation	Critique	Cooperation
Financial Crisis	-0.04	0.03	-0.02	-0.01
	(0.05)	(0.03)	(0.03)	(0.02)
Chinn-Ito Index	-0.07	0.03	0.15***	-0.13***
	(0.07)	(0.05)	(0.05)	(0.04)
IMF, WB, GATT/WTO member	0.04	0.06	0.02	-0.14***
	(0.07)	(0.04)	(0.05)	(0.03)
KOF trade globalization/10	-0.04	0.03***	0.00	-0.02
	(0.03)	(0.01)	(0.02)	(0.01)
GDP per capita (natural log)	0.02	0.03	0.03	0.02
	(0.06)	(0.04)	(0.04)	(0.03)
Liberal democracy index	0.12	-0.32***	0.00	0.29***
	(0.12)	(0.12)	(0.09)	(0.10)
Left executive	-0.01	0.05*	0.01	-0.04*
	(0.04)	(0.03)	(0.03)	(0.02)
Right executive	0.00	-0.01	0.04	-0.02
	(0.04)	(0.03)	(0.03)	(0.02)
Constant	-0.01	-0.06	-0.26	0.44
	(0.48)	(0.31)	(0.34)	(0.27)
Observations	1,976	3,409	1,976	3,409
R-squared	0.16	0.09	0.05	0.05
Number of CIDnum	113	143	113	143

A3.6. Table including IMF/World Bank program participation

VARIABLES	(1)<br>ECONscale	(2)<br>ECONscale
Financial Crisis	0.03	-0.04
	(0.07)	(0.05)
IMFprogram	0.00	-0.02
	(0.04)	(0.03)
WBprogram	-0.07	0.01
	(0.06)	(0.04)
Chinn-Ito Index	0.39***	-0.09
	(0.10)	(0.08)
KOF trade globalization/10	0.05	-0.06***
	(0.04)	(0.02)
GDP per capita (natural log)	0.07	-0.00
	(0.09)	(0.06)
Liberal democracy index	0.07	0.58***
	(0.19)	(0.20)
Left executive	0.05	-0.13***
	(0.06)	(0.05)
Right executive	0.03	-0.03
	(0.06)	(0.05)
Constant	-0.85	0.41
	(0.75)	(0.53)
Observations	1,976	3,273
R-squared	0.14	0.04
Number of CIDnum	113	143

A3.7. Changes in average financial and trade openness over time

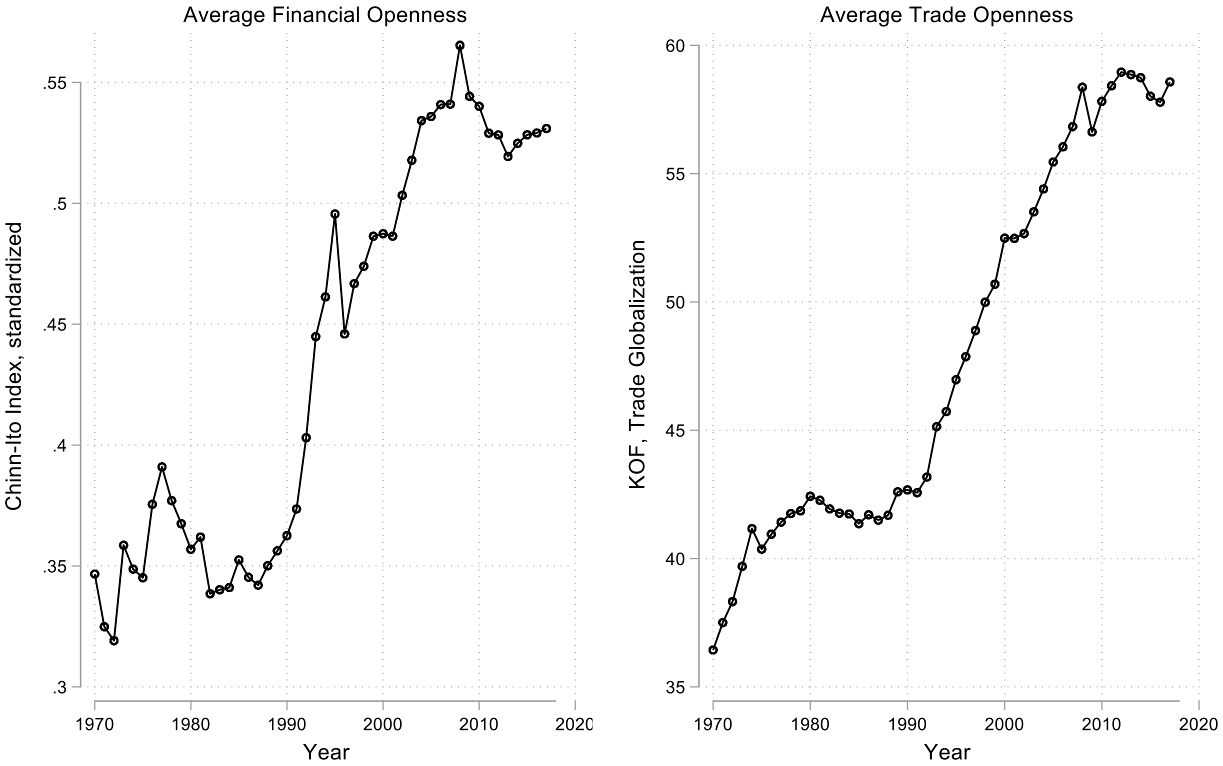

Supplement: Supplementary file 5 — (PDF 471 kb) [file 11558_2020_9404_MOESM5_ESM.pdf]
